# Economic Evaluation of Brief Psychodynamic Interpersonal Therapy in Patients with Multisomatoform Disorder

**DOI:** 10.1371/journal.pone.0083894

**Published:** 2014-01-22

**Authors:** Nadja Chernyak, Heribert Sattel, Marsel Scheer, Christina Baechle, Johannes Kruse, Peter Henningsen, Andrea Icks

**Affiliations:** 1 Department of Public Health, Faculty of Medicine, Heinrich-Heine University Duesseldorf; German Diabetes Center, Institute of Biometrics and Epidemiology, Duesseldorf, Germany; 2 Department of Psychosomatic Medicine and Psychotherapy: Klinikum rechts der Isar, Technical University Munich, Munich, Germany; 3 German Diabetes Center, Institute of Biometrics and Epidemiology, Duesseldorf, Germany; 4 Department of Psychosomatic Medicine, University of Düsseldorf, and Centre for Psychosomatic Medicine, Justus Liebig University of Giessen, Giessen, Germany; University of Western Brittany, France

## Abstract

**Background:**

A brief psychodynamic interpersonal therapy (PIT) in patients with multisomatoform disorder has been recently shown to improve health-related quality of life.

**Aims:**

To assess cost-effectiveness of PIT compared to enhanced medical care in patients with multisomatoform disorder.

**Method:**

An economic evaluation alongside a randomised controlled trial (International Standard Randomised Controlled Trial Number ISRCTN23215121) conducted in 6 German academic outpatient centres was performed. Incremental cost-effectiveness ratio (ICER) was calculated from the statutory health insurance perspective on the basis of quality adjusted life years (QALYs) gained at 12 months. Uncertainty surrounding the cost-effectiveness of PIT was presented by means of a cost-effectiveness acceptability curve.

**Results:**

Based on the complete-case analysis ICER was 41840 Euro per QALY. The results did not change greatly with the use of multiple imputation (ICER = 44222) and last observation carried forward (LOCF) approach to missing data (ICER = 46663). The probability of PIT being cost-effective exceeded 50% for thresholds of willingness to pay over 35 thousand Euros per QALY.

**Conclusions:**

Cost-effectiveness of PIT is highly uncertain for thresholds of willingness to pay under 35 thousand Euros per QALY.

## Introduction

Patients with multisomatoform disorder (MSD) are characterized by several medically unexplained somatic symptoms. They have significant functional impairment, are difficult to treat [1] and show high health care utilization rates [2]. Against this background a large, multi-centre, randomised controlled trial was conducted in Germany to test the efficacy of a brief psychodynamic-interpersonal psychotherapy (PIT) in patients with MSD. According to this study [3], PIT improved patient quality of life measured by the SF-36 physical component summary score (PCS) at nine months after the end of the treatment significantly better than a control intervention – enhanced medical care (EMC). Since PIT has higher treatment costs compared to the control intervention, the question of cost-effectiveness arises. Building on the results of the trial, the relative efficiency of the PIT compared to EMC was analysed from the perspective of the statutory health insurance. In the following, design and results of the trial-based economic evaluation are reported and discussed.

## Methods

### Ethics Statement

Ethic committees of the medical faculties of Technical University München, Heinrich–Heine University Düsseldorf, University Heidelberg, University Regensburg, Wilhelms University Münster, the ethic committee of Medical Association Westfalen-Lippe, and the ethic committee of the Medical University Hannover approved the study. Written informed consent was obtained from all study participants.

### Clinical trial

Full details of the study have been described elsewhere [3]. The protocol for this trial is available as supporting information (see Protocol S1). Briefly, the study was conducted at six university departments of psychosomatic medicine in Germany (Munich, Düsseldorf, Heidelberg, Hannover, Münster and Regensburg). Two hundred and eleven patients aged 18–77 years who have had multisomatoform disorder according to established criteria [4] were recruited from the outpatient departments of neurology and internal medicine as well as pain treatment centres and an orthopedics private practice. The independent clinical trials unit at the University of Düsseldorf stored all the data, regularly monitored all project sites and analyzed the primary and secondary outcome data.

**Figure 1:**
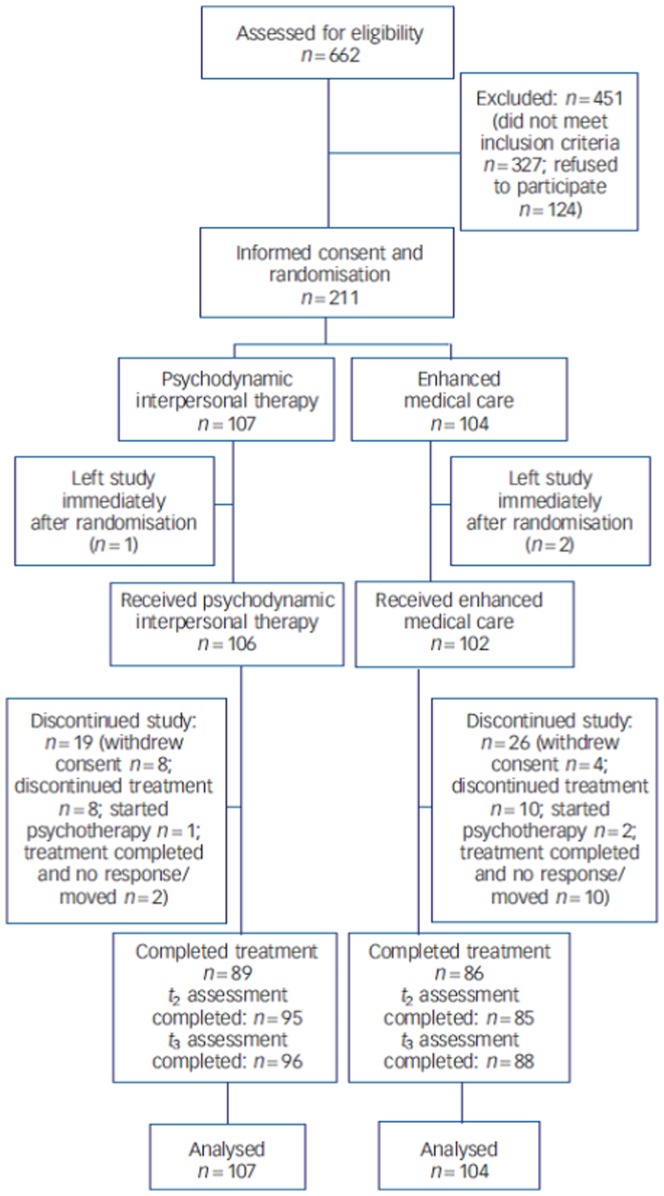
Consort chart of Patients with Multisomatoform Disorder in a Trial of Short-Term Psychodynamic Interpersonal Therapy.

The patients were randomized to receive either twelve weekly sessions of PIT (intervention group, N = 107), or three sessions of EMC (control group, N = 104), see Fig. 1. The intervention consisted of one session of PIT during 12 weeks – specifically adapted to the needs of patients in bodily distress. The first session lasted up to 90 minutes; all other sessions were approximately 45 minutes. The participants were treated in the outpatient departments of psychosomatic medicine. Patients in the EMC group had three approximately 30-min sessions at six-week intervals delivered by physicians at the referring outpatient departments specifically trained in EMC. Patients in this group received counseling regarding the therapeutic options based on the national evidence-based guidelines for the treatment of somatoform disorders/functional somatic syndromes in primary and somatic specialist care. At the end of the therapy, the therapists delivering EMC recommended – if necessary – additional psychotherapeutic or somatic treatments and medication for the patients in a comparable manner as in the PIT group.

The primary outcome of the trial was the physical component summary score (PCS) of the Short Form Health Survey (SF-36). As the sustainability of potential treatment effects is particularly important in a chronic condition like multisomatoform disorder, improvement was measured nine month after the end of the treatment. Follow-up assessment questionnaires were sent and returned by post.

### Economic evaluation

To determine relative efficiency of the PIT, an incremental cost-effectiveness ratio (ICER), i.e. the ratio of the difference in mean costs divided by the difference in mean effects between the PIT and the EMC group was estimated. The analysis was performed from the perspective of the statutory health insurance. Since the evaluation covered only one year alongside the trial, costs and effects were not discounted.

#### Effects

In the clinical trial the improvement of quality of life was measured by the physical component summary score (PCS) of the SF-36, one of the most widely used generic profile-based patient-reported outcome measures (PROMs). Whereas profile-based PROMS can be very informative in cases where the end point of interest is a change in specific dimensions of health, they are not suitable for economic evaluation of health care interventions. There are two main reasons for this. First, the profile scores (e.g. SF-36 dimension scores) usually do not have interval properties (i.e. where the scores represent equal intervals) and thus the cost-effectiveness ratios are likely to be meaningless [5]. Second, profile-based PROMs do not factor individual preferences in their measurements of health; therefore, there is no evidence that higher scores necessarily represent the most preferred outcome [6]. Hence, for the purposes of economic analysis, health improvement was measured in terms of quality adjusted life years (QALYs) gained. QALYs summarize health into a single index, consider individual preferences and are assumed to have interval properties. They are calculated as the product of a preference for a particular health state and duration of this health state. Preferences for a particular health state are measured on a scale from 0 to 1, where 0 and 1 represent death and full health, respectively [7]. Separate measures are available to capture preferences for health states. In this study we used SF-6D [8] that derives preference-based scores from the SF-36 by using population-based preferences (utilities) for the SF-36 health states. Preferences were calculated from the SF-36 data collected at baseline and at a 1 year follow-up (nine months after the end of the treatment). QALYs gained per patient over the trial period in each group were calculated using linear interpolation between measurement points and calculating the area under the curve [7].

#### Costs

Only direct treatment costs, i.e. resource use directly associated with PIT and EMC from the statutory health insurance perspective were compared between both groups. The number of actually attended sessions, documented by therapists, was used to calculate treatment costs: time spent per session in PIT and EMC groups was monetary valued using the reimbursement rate of 80 Euro per 45 min PIT session and 54 Euro per 30 min EMC session (Bavarian schedule of fees; http://www.aok-gesundheitspartner.de/by/arztundpraxis/vertraege/index_02844.html, last viewed 01.03.2012).

#### Statistical analysis

Statistical analyses were based on the intention-to-treat approach. Data on treatment cost were available for all trial participants. However, 10% and 15% of the patients in the PIT and EMC group, respectively, did not provide 12 months follow up data necessary to calculate utility weights for QALYs. In a base-case evaluation complete case analysis was performed to estimate the difference in costs and outcomes between the PIT and the EMC and to calculate the incremental cost-effectiveness ratio. Mean difference in effects between groups and 95% confidence intervals were obtained by a bootstrap procedure (5000 replications).

To represent uncertainty surrounding the cost-effectiveness of PIT, cost-effectiveness acceptability curve (CEAC) was used as an alternative to confidence intervals around the ICER. CEAC shows the probability of the intervention being cost-effective for different threshold values of willingness to pay for a QALY gained [9]. The non-parametric bootstrap method was used to construct the CEAC. Five thousand replicated data sets were generated to calculate the proportion of replications where PIT had positive incremental monetary benefit (ICER was below a particular threshold value of willingness to pay). This was done for different threshold values of willingness to pay.

#### Sensitivity analyses

In the base-case evaluation cases with missing SF-36 data were excluded. Two other approaches to handle missing data – last observation carried forward (LOCF) and imputation – were examined in sensitivity analyses. The imputation of missing data was performed by using Multivariate Imputation by Chained Equations [10].

## Results

Seventeen percent of the PIT group and 16% of the EMC group did not visit all scheduled sessions. The mean number of contacts and the associated costs were significantly higher in the PIT group than in the EMC group (893 and 141 Euro respectively) with difference in mean costs between interventions accounting for 752 Euro. Difference in mean QALYs gained over 12 months was 0.02, with a 95% CI of −0.01 to 0.05, indicating non-significance. Utility scores at baseline and at nine months follow up and QALYs gained per group are reported in the Table 1.

**Table 1:** Utility scores at baseline and nine months follow up and QALYs gained per group.

	PIT	EMC
	Mean (sd)	Mean (sd)
SF 6D scores at baseline	0.50 (0.09)	0.51 (0.10)
SF 6D scores 9 month follow up	0.59 (0.14)	0.55 (0.13)
QALYs gained	0.55 (0.10)	0.53 (0.11)

The mean incremental cost-effectiveness ratio (ICER) was 41840 Euro per QALY gained. The results for ICER did not change greatly with the use of imputed full sample data (ICER = 44222) as well as with LOCF approach to missing data (ICER = 46663).

**Figure 2:**
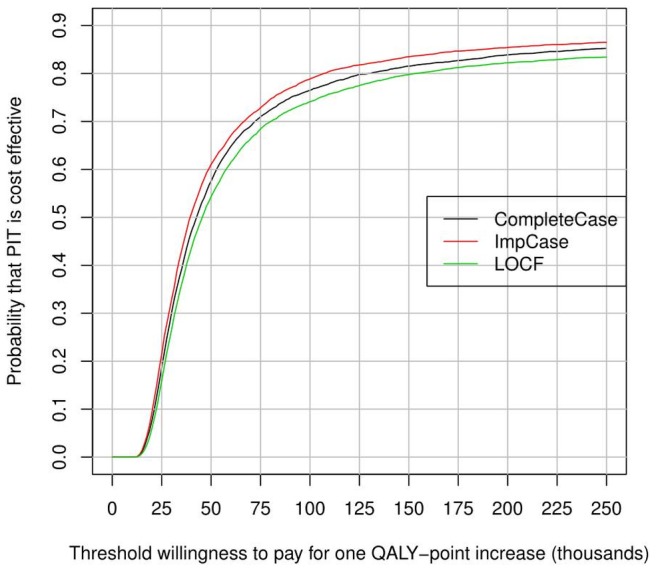
Cost- effectiveness acceptability curves for Psychodynamic Interpersonal Therapy (PIT).

Cost-effectiveness acceptability curves are shown in Figure 2. The probability of PIT being cost-effective grew as the threshold willingness to pay per QALY gained increased. The probability of PIT being cost-effective exceeded 50% for willingness to pay levels higher than 35 thousand Euros per QALY.

## Discussion

We evaluated cost-effectiveness of a psychodynamic interpersonal therapy (PIT) compared to enhanced medical care in patients with multisomatoforme disorder using QALYs as an outcome for an economic analysis. In order to calculate QALYs, preference-based measures of health state are necessary. Separate measures are available for this purpose, and there is no consensus on which measure is best. We used SF-6D [8] that derives preference-based scores from the SF-36 data by using population-based preferences (utilities) for the SF-36 health states. Using this approach, the difference in mean QALYs between treatment groups was not statistically significant, although statistically significant difference between PIT and EMC groups was shown for the physical component score of the SF-36. PIT improved patient quality of life at nine months after the end of the treatment better than EMC (mean improvement of PCS: PIT 5.3; EMC 2.2), with a small to medium between-group effect size (d = 0.42; CI: 0.15–0.69, p = 0.001). However, no significant difference was found for the mental component score [3]. There are several factors contributing to a higher uncertainty of the intervention effect when QALYs are used as an outcome measure. First, the SF-6D health state classification has compromised the descriptive richness of the original SF-36, as it is derived from the SF-36 by reducing its size (11 items) and simplifying its structure (6 instead of 8 dimensions). SF-6D scores have been shown to be less sensitive to group differences and less responsive to changes in health over time compared to the SF-36 scales [11]. Hence, the PCS score reflecting the change in a specific dimension of health was more sensitive than the SF-6D index reflecting the strength of people's preferences for different aspects of health, including mental health. Second, the SF-6D derives preference-based scores from the SF-36 by using preferences for the SF-36 health states from the *general population* rather than *patient* preferences. Although use of preferences from the general population is the recommended practice for cost-effectiveness analysis, these preferences may be different from those of patients experiencing particular health states and this discrepancy could also account for the lower responsiveness to changes in health.

The lack of statistical significance for difference in QALYs between treatment groups complicates the estimation of the ICER and interpretation of uncertainty related to it: cost-effectiveness acceptability curve (CEAC) based on bootstrapping replications had to be used as an alternative to confidence intervals around the ICER. However, also the inference approach, i.e. estimating the sampling distribution of an incremental cost-effectiveness ratio has limitations [12]. In particular, it could lead to an eventual rejection of potentially beneficial new intervention. Hence, we report ICER for PIT compared to EMC based on differences in mean costs and outcomes and show the probability of PIT being cost-effective for various thresholds of willingness to pay per QALY gained using the concept of cost-effectiveness acceptability curve in order to explore decision uncertainty.

The results of the complete case analysis (CCA), which was applied in the base-case evaluation, can be biased if the complete cases systematically differ from the original sample (when the missing information is not missing completely at random). We decided to apply CCA, because it is considered to be an acceptable method with small amounts of missing information [13] and other methods of handling missing data have their limitations too. The results for the ICER did not change greatly with the use of imputed full sample data (ICER = 44222) as well as with LOCF approach to missing data (ICER = 46663). Hence, the results of the CCA are unlikely to be largely biased.

### Limitations of the study

Preferences for health states were derived from the SF-36 using scoring algorithm which is based on health state preferences of the UK general population. Hence, preferences of German general population were not considered in our analysis. The main limitation of the study was that we were unable to consider health care utilization not directly related to the intervention (received outside the intervention) in our analysis. In principle, health care received outside the intervention should be incorporated into the calculation of ICER, because it may change as a result of the intervention and also influence the amount of QALYs gained in different intervention groups. In practice, however, it is often impossible to collect such data in a reliable and valid manner. We could not collect trustworthy health care utilization data for the whole duration of the study because self-report was the only available data source and we do not consider it to be valid for the follow-up period of 9 months after the end of treatment because of recall bias. Future studies of cost-effectiveness of PIT should try to collect valid data on general health care utilization.

### Conclusions and needs for future research

Our results suggest that cost-effectiveness of PIT is highly uncertain for thresholds of willingness to pay under 35 thousand Euros per QALY. Larger trials would be needed to reinforce the power of economic analyses calculating QALYs on the basis of the SF-6D index and to reduce decision uncertainty with regard to the cost-effectiveness of PIT.

As we did not analyse the impact of PIT on utilization of other health care services, our estimation of the ICER is conservative. PIT may be also more cost-effective in the long term if the effect of experimental intervention lasts longer (e.g. due to an increase in specific interpersonal and health-related self-efficacy).

## Supporting Information

Protocol S1 **PISO clinical trial protocol.**
(PDF)
