# Supplementary material for: Economic Evaluation of Brief Psychodynamic Interpersonal Therapy in Patients with Multisomatoform Disorder
Source: PLoS One. 2014 Jan 22;9(1):e83894. doi: 10.1371/journal.pone.0083894 (PMC3898909; doi:10.1371/journal.pone.0083894)
Supplement: Protocol S1 — PISO clinical trial protocol. (PDF) [file pone.0083894.s001.pdf]

PISO: Clinical Trial Protocol

Table of Contents

**Protocol Summary**		**2**
**1.**	**General Information**	**3**
**2.**	**Background Information**	**4**
**3.**	**Trial objectives and purpose**	**6**
**4.**	**Trial design**	**7**
**5.**	**Selection and withdrawal of subjects**	**10**
**6.**	**Treatment of subjects**	**10**
**7.**	**Assessment of efficacy**	**11**
**8.**	**Assessment of safety**	**11**
**9.**	**Statistics**	**13**
**10.**	**Direct access to source data/ documents**	**16**
**11.**	**Quality control and quality assurance**	**16**
**12.**	**Ethics**	**17**
**13.**	**Data Handling and Record Keeping**	**18**
**14.**	**Insurance**	**19**
**15.**	**Publication Policy**	**19**
**16.**	**Supplements**	**20**
	**Supplement 1: Investigators and Trial Sites**	**20**
	**Supplement 2: Trial Diagram**	**24**
	**Supplement 3a: PISO Treatment Manual , abbreviated English version**	**25**
	**Supplement 3b: Control Group Intervention: Guideline Enhanced Medical Care**	**31**
	**Supplement 4: Draft Patient Information and Consent Forms**	**33**
	**Supplement 5: Methods for psychophysiological measurements**	**40**
	**Supplement 6: References**	**41**

Protocol Summary

**Title of trial**	Psychosomatic Intervention for Patients with MultiSOmatoform Disorder in Different Somatic Specialities
**(Acronym)**	PISO
**Disease or condition**	Multisomatoform Disorder (enduring medically unexplained bodily complaints)
**Participants**	Patients with a Multisomatoform Disorder in different somatic specialities
**Design / methodology**	Multi-centre two-arm randomized controlled trial with major end point at 1-year follow-up, the guidelines of Good Clinical Practice (GCP) will be followed, reporting will follow the CONSORT rules
**Topic / objectives / aim**	Patients with medically unexplained physical symptoms are „high utilizers“ of the health care system with high psychiatric co-morbidity and severe impairments in quality of life (QoL). There is preliminary evidence that psycho-dynamic-interpersonal therapy (PIT) is beneficial as it reduces the intensity of physical symptoms and increases quality of life. The trial interventions so far lacked generalizability over a larger clinical spectrum of disabling somatoform symptoms. We developed a multi-centre two-arm randomized controlled trial with a primary end point and follow-up after one year. PISO has two new aspects:<br>- PISO uses a diagnostic category that is independent of the type of currently dominant symptom and therefore serves as a common point of reference.<br>- PISO uses a manualized psychotherapeutic intervention that is adapted to the specific lead symptom in the beginning, but later on emphasizes more general aspects of experiencing "unexplained" physical symptoms across single functional syndromes and somatic specialities.<br>In the trial, we test a bio-psycho-social model of change including psychobiological parameters like heart rate variability and prefrontal/ limbic neural activations. If the intervention tested in PISO proves to be efficacious as compared to enhanced medical care it will be useful as an economic and versatile tool that is applicable in cooperation with psychosomatic medicine across a range of somatic specialities.
**Eligibility criteria - inclusion**	Patients screened positive and diagnosed in a structured interview in different somatic specialities with a diagnosis of pain-predominant multi-somatoform disorder and a QoL of 1 standard deviation below population norm in the SF-36, signed informed consent
**Eligibility criteria - exclusion**	Age younger than 18 years, insufficient German language ability, insufficient cognitive abilities (Mini Mental State < 24), severe and chronic somatic disease, severe co-morbid mental disorder causing major impairment of social functioning
**Groups / interventions**	Experimental intervention: Manualized phase – defined 12 session intervention based on the principles of psychodynamic-interpersonal psychotherapy.<br>Control intervention: Enhanced medical care of the symptom leading to current health care utilisation
**Endpoints / primary outcome(s)**	SF-36 Physical Component Summary (PCS): In patients with multi-somatoform disorder, symptom-related incapacity is best captured with the PCS. The German version of the SF-36 Health survey short form has been validated and is sensitive to change, the assumed effect size of 0.50 is clinically relevant.<br>(SF-36 PCS popul: 50,0, SD 10; popul. w. somatoform disorders: 45.8, SD 10)
**Sample size**	Calculated as n = 2 x 88 (n = 88 PISO-intervention, n = 88 control intervention)
**Trial duration**	36 months: Trial preparation and training of therapists month 0-5, Screening and recruitment month 5-16, Intervention month 6-19, Follow-up month 20-31, data analysis and publication months 32-36
**Follow-up**	Final assessment at 3 months and Follow up at 1 year
**Principal / coordinating investigator**	Peter Henningsen, Priv. Doz. Dr. med., Deputy Head
**PI / CI Address**<br>**Street**<br>**Zip City/town**<br>**Tel**<br>**Fax**<br>**E-mail**	University Hospital Heidelberg, Dept. of Psychosomatic Medicine<br>Thibautstr.2<br>D-69115 Heidelberg, Germany<br>**(Note: new contact data from Sept. 2005, see Trial Protocol)**<br>+49-6221-56-5865<br>+49-6221-56-33586<br>peter.henningsen@med.uni-heidelberg.de

1. General Information

1.1 Title of the Protocol

Psychosomatic Intervention for Patients with Multisomatoform Disorder in Different Somatic Specialities
Acronym: PISO **DFG/BMBF Code**: He 3200/4-1; 60665-02-2/167/04

1.2 Sponsor

Technical University Munich, University Hospital "Rechts der Isar"
(Note: the Coordinating Investigator will become Head of the Dept. of Psychosomatic Medicine in Munich from September 2005)

1.3 Name and title of the person(s) authorized to sign the protocol and the protocol amendment(s) for the sponsor

Claus Thaller**,** Head of Administation, University Hospital "Rechts der Isar", Technical University Munich

1.4 Name and title of the investigators who are responsible for conducting the trial, and addresses and telephone numbers of the trial sites.

- Priv.Doz. Dr.med. Peter Henningsen, Coordinating Investigator,
currently:Dept of Psychosomatics, University Hospital Heidelberg, Thibautstr. 2, 69115 Heidelberg; Tel. +49-6221-56-5865; Fax +49-6221-56-33586, peter.henningsen@med.uni-heidelberg.de
From Sept. 2005: Dept. of Psychosomatics, University Hospital "Rechts der Isar", Langerstr. 3, 81675 Munich; Tel. +49-89-4140-4310; p.henningsen@tum.de
(As this change of affiliation is within the PISO network, efficient conduct of the trial is assured. Heidelberg will remain a valid center with investigators responsible for correct recruitment etc.)

- Prof. Dr. rer.nat. C. Ohmann, Data Management, Monitoring, Biostatistics,
Coordinating Center for Clinical Trials, University of Düsseldorf, Moorenstr. 5, 40225 Düsseldorf, Tel. +49-211-811-9700; Fax +49-211-811-9702, ohmannch@uni-duesseldorf.de

Complete List of Investigators, Cooperating Physicians and Trial Sites see:
16. Supplements, Supplement 1: Investigators and Trial Sites

1.5 Name, title, address, and telephone number(s) of the qualified physician who is responsible for all trial-site related medical decisions (if other than investigator)

Does not apply, as local investigators are responsible for trial-site related medical decisions.

2. Background Information

2.1. Name and description of the intervention

Psychodynamic-interpersonal psychotherapy for patients with pain predominant multisomatoform disorder in different somatic specialities

PISO will test a specific form of psychotherapy which is adapted to the needs of patients who complain persistently about bodily symptoms like pain and bodily dysfunction for which no adequate organic explanation can be found. These patients typically turn for help to somatic specialists like pain treatment centers run by anaesthetists or orthopaedic surgeons, departments of neurology or internal medicine. Typically they reject psychosocial explanations for their symptoms and therefore are not motivated to enter into classical psychotherapy. Hence psychotherapeutic strategies have to be developed that are acceptable to this patient group as a whole and not only to the few selected ones who are motivated enough intrinsically or extrinsically to seek help in a psychotherapeutic/ psychosomatic/ psychiatric institution on their own.

The intervention tested in PISO is based on the principles of short term psychodynamic-interpersonal psychotherapy (PIT). This is a modern type of psychodynamic psychotherapy that has been shown to be effective in different populations and settings (Leichsenring et al. 2004). The specific application to patients with somatoform disorders is based on adaptations of psychodynamic-interpersonal therapy as developed by Guthrie in the UK and Rudolf and Henningsen (2003) in Germany.

2.2. Summary of findings from preclinical studies and previous clinical trials/ studies/ clinical standards

Three of the investigators, with the coordinating investigator as principal author, were members of the group who established evidence-based practice guidelines on the diagnosis and treatment of somatoform disorders for the German Association of scientific medical societies (AWMF). These guidelines won the DKV-Cochrane prize (€ 25.000.-) in 2000 for distinguished contributions to evidence based medicine. Updated guidelines were published in 2002 (Henningsen et al. 2002). The guidelines are based on a systematic review of the evidence base for the treatment of somatoform disorders. More than 3000 articles were screened and the relevant primary studies were extracted in tables. The literature that appeared since 2002 was searched systematically in November/ December 2004 using the same search strategy that was used for the guidelines and the first update.

Based on these data psychotherapy has been shown to be effective in so-called functional somatic syndromes, with best evidence being available for Cognitive-Behavioural Therapy for Chronic Fatigue Syndrome and Hypochondriasis and for Psychodynamic-Interpersonal Therapy for functional gastrointestinal disorders. Effectiveness in these studies relates to reductions in the intensity of physical symptoms and improvements in quality of life.

However, two problems currently limit the validity and usefulness of treatments in this field:

On the one hand, patients with medically unexplained bodily symptoms typically see themselves as physically ill and when being sent to a psychiatrist/ psychotherapist or being offered usual psychotherapy, only a selected sub-group will accept this.

On the other hand, trials that address unselected patients directly in medical specialities have so far been confined to examining efficacy and effectiveness in specific functional somatic syndromes (FSS) like irritable bowel syndrome or fibromyalgia only. This may not be the best paradigm for evaluating the effectiveness of such treatments in somatization, as the evidence for specific discrete FSS is weak. They display a wide overlap of symptoms and share many non-symptom characteristics like association with depression and anxiety and similar response to non-pharmacologic and pharmacologic treatments (Wessely et al. 1999, Barsky and Borus 1999).

2.3. Risks and benefits, if any, to human subjects

Benefits

According to our main hypothesis patients from somatic speciality clinics who fulfill diagnostic criteria for multisomatoform disorder with predominant pain symptoms and who receive manualized psychotherapy based on PIT principles will show improved quality of life (QoL) in comparison to controls who receive enhanced medical care. Furthermore, according to the secondary hypothesis on a psychobehavioural level, patients with multisomatoform disorder in somatic speciality clinics who receive manualized psychotherapy will show reduced health care utilisation, less physical symptoms and more adaptive illness representations and higher levels of emotional awareness compared to controls.

Risks

As it is based on common therapeutic strategies in patients with medically unexplained symptoms, the intervention in PISO is not experimental. Psychotherapeutic interventions like this have repeatedly been shown to be relatively safe, but all have potential side effects. In particular, there is a risk that during the intervention patients may transiently become more depressive and/ or suicidal as a sign of altered emotional processing of life stresses. Close monitoring of this risk by the therapist is assured through the PIT manual and is helped by the fact that the intervention is taking place not in group, but in single patient format. Appropriate action (e.g. antidepressive medication, psychiatric in-patient referral) is defined in a manual for clinical care that will be adapted to the local situation of each centre.

2.4. Description of and justification for the route of administration/ method/ intervention

When left untreated, a majority of frequently attending patients with medically unexplained symptoms continues to be incapacitated by their symptoms even after three years (Gureje and Simon 1999, Reid et al. 2003). The evidence base for the effectiveness of other, in particular psychopharmacological, treatments is weaker than for psychotherapeutic ones.
A systematic evaluation of manualized short-term PIT which is aimed at patients in somatic speciality settings, transcends the diagnostic constraints of single functional somatic syndromes and allows easy transfer to daily clinical praxis is lacking up to now.

2.5. Statement that the trial will be conducted in compliance with the protocol, GCP and the applicable regulatory requirement(s).

PISO will be conducted in compliance with the protocol, GCP and the applicable regulatory requirement(s).

2.6. Description of the population to be studied

The PISO trial focuses on patients with pain predominant multisomatoform disorder. Multisomatoform disorder (MSD) is a diagnostic construct proposed by Kroenke et al. (1997) for a moderate severe disorder of three current and a history of at least two years of organically unexplained bodily symptoms and severely reduced quality of life that appears to be more appropriate to describe patients with medically unexplained bodily symptoms than other diagnoses like somatization disorder. Empirically, pain is the most frequent type of complaint, followed by functional complaints and fatigue.
Patients with these symptoms typically see themselves as physically ill and therefore tend to consult somatic specialists like neurologists, pain therapists or specialists for internal medicine. Taking this into account, patients with multisomatoform disorder will be recruited in different somatic speciality wards or outpatient clinics, thereby avoiding the selection effects which are operative when treating patients who come to psychosomatic/ psychiatric departments directly. Therapy will be performed as outpatient treatment only.

2.7 References to literature and data that are relevant to the trial, and that provide background for the trial

Guthrie E, Moorey J, Margison F et al. Cost-effectiveness of brief psychodynamic-interpersonal therapy in high utilizers of psychiatric services. Arch Gen Psychiatry 1999; 56: 519-526.

Hamilton J, Guthrie E, Creed F et al. A randomized controlled trial of psychotherapy in patients with chronic functional dyspepsia. Gastroenterology 2000; 119: 661-669.

Henningsen P, Hartkamp N, Loew T, Sack M, Scheidt CE. Somatoforme Störungen - Leitlinien und Quellentexte. Stuttgart: Schattauer, 2002.

Kroenke K, Spitzer RL, deGruy FV et al. Multisomatoform disorder. An alternative to undifferentiated somatoform disorder for the somatizing patient in primary care. Arch.Gen.Psychiatry 1997; 54: 352-358.

Leichsenring F, Rabung S, Leibing E. The efficacy of short-term psychodynamic psychotherapy in specific psychiatric disorders: a meta-analysis. Arch Gen Psychiatry 2004; 61:1208-16.

Margison F. Psychodynamic-interpersonal therapy. Oxford: Oxford University Press, 2002.

Reid S, Crayford T, Patel A, Wessely S, Hotopf M. Frequent attenders in secondary care: a 3-year follow-up study of patients with medically unexplained symptoms. Psychol Med 2003; 33: 519-524.

Rief W, Henningsen P. Somatoforme Störungen [Somatoform Disorders]. In Senf W, Broda M (ed.): Praxis der Psychotherapie 3rd ed. Ch. X/36. Stuttgart: Thieme 2005, p 529-544.

Rudolf G, Henningsen P. Die psychotherapeutische Behandlung somatoformer Störungen [Psychotherapeutic treatment of somatoform disorders]. Z Psychosom Med Psychotherap 2003; 49: 3-19

3. Trial Objectives and Purpose

Severe symptoms of pain and bodily dysfunction that remain medically unexplained after appropriate investigation are very frequent in primary care and in most medical speciality clinics, with frequencies of between 30-40% for gastroenterology and neurology and up to 66% for gynaecology (Nimnuan et al. 2001, Reid et al. 2001). In the German general population, lifetime prevalence for pain-dominant somatoform disorders is 33.7%, for those that significantly impair functioning it is 12.3% (Grabe et al. 2003). Psychiatric morbidity is high, and many patients have chronic psychosocial problems. The dysfunctional behaviour of such patients is often maintained by fixed illness representations that signify organic disease despite reassurance to the contrary by multiple physicians. Patients with medically unexplained bodily symptoms are so-called “high utilizers” of the health care system, incurring costs that are up to 10 times higher than in average patients (Bass 2001). Even patients not fulfilling diagnostic criteria for somatization disorder proper are severely functionally disabled by their organically unexplained symptoms (Kroenke et al. 1997, Dickinson et al. 2003). When left untreated, a majority of frequently attending patients with medically unexplained symptoms continues to be incapacitated by their symptoms even after three years (Gureje and Simon 1999, Reid et al. 2003).
Multisomatoform disorder (MSD), a diagnostic construct proposed by Kroenke et al. (1997) for a moderate severe disorder of three current, a history of at least two years of organically unexplained bodily symptoms and severely reduced quality of life appears to be more appropriate to describe these patients. In concentrating on current bodily symptoms, this diagnostic construct avoids the instability demonstrated in somatization diagnoses that are based on lifetime symptoms like somatization disorder (SD) or abridged somatization disorder (ASD) (Dickinson et al. 2003).

Building on our previous work we want to a) examine the effectiveness of PIT in patients with pain predominant multisomatoform disorder in different somatic specialities like anaesthesiological pain treatment centres, neurological, dental and internal medicine departments and b) test a biopsychosocial model of change in which improvements particularly in disability and physical symptoms will be mediated by changes in illness representations and in heart rate variability in a stress-task paradigm.

4. Trial design

4.1. Primary and secondary endpoint

Primary endpoint

The primary purpose of this study is to assess if patients who fulfil diagnostic criteria from multisomatoform disorder with predominant pain symptoms and who receive manualized psychotherapy based on PIT principles (test group) will or will not show improved quality of life in comparison to controls who receive enhanced medical care (control group). The primary analysis will be based on the German version of the SF-36 Health survey short form (Bullinger and Kirchberger 1998). The statistical hypothesis will be based on the Physical Component Score of SF- 36. (t 0, t 1, t 2)

Statistical hypothesis:

H_0: *no treatment difference between control and test group*
vs.
H_1: *difference between control and test group*

Secondary endpoints

- **IPQ, Brief Form**
  The brief form of the „Illness Perception Questionnaire“ (BIPQ, Broadbent et al. in submission) provides a quantitative measure of the components of illness representations (identity, consequences, timeline, personal control, treatment control, emotional representations, illness coherence, psychological attributions, risk factor attributions) that has been shown in treatment trials to be sensitive to change. The German version was developed and validated by Gaab (unpublished manuscript) (t 0, t 1, t 2).
- **PHQ-D**
  The „Patient Health Questionnaire”, German version (PHQ-D) is a brief self-report measure for anxiety, depression and other mental disorders. Criterion validity was established with respect to diagnostic gold standards and the PHQ has proven to be a responsive and reliable measure of depression treatment outcome (Löwe et al. 2004) (t 0, t 1, t 2).
- **SOMS-7**
  Seven-day version of the „Screening for Somatoform Symptoms“. A 53-item instrument for the evaluation of treatment effects in somatoform disorders, covering all somatic symptoms occurring in somatization disorder, according to DSM-IV and ICD-10 (Rief and Hiller 2003)(t 0, t 1, t 2).
- **Whiteley-Index-7**
  The seven-item version of the Whiteley Index, an established hypochondriasis scale first described by Pilowsky, was developed and validated by Christensen et al. (2003) specifically to assess treatment effects. (t 0, t 1, t 2)
- **TAS**
  The “Toronto Alexithymia Scale” (Bagby et al. 1994) is a 20-item self-report measure of alexithymia with good internal consistency and test-retest reliability and a factor structure congruent with the alexithymia construct (t0). Alexithymia is conceptually related to expected effects of the treatment intervention and will be therefore assessed as a predictor variable.
- **ECR**
  The Questionnaire “Experiences in Close Relationships” (ECR, Brennan et al. 1998, german adaption: the Bochum Adult Attachment Questionnaire, Neumann et al., submitted) measures the two attachment dimensions avoidance and anxiety to characterize the quality of partner attachment (t0).
- **HAQ**
  The “Helpful Alliance Questionnaire” (Luborsky 1984, German Version: Bassler et al. 1995) is a brief (12 Items) questionnaire for the assessment of perceived therapeutic relationship and therapeutic process. It will be answered as well by the patient as by the therapist and allows interpretation of potentially relevant moderators of therapy outcome (t1).
- **Heart rate variability**
  HRV measurement and analysis equipment will be present at each centre. Time-domain (RMSSD) and frequency domain (HF-HRV) analysis will be performed during a stress test

with the emotional stroop paradigm (detailed description of methods see Supplement 5). (t 0, t 1, t 2)

4.2 Description of the type/design of trial to be conducted

Multi-centre two-arm randomized controlled trial with major end point at follow-up at 1 year, the guidelines of Good Clinical Practice (GCP) will be followed, reporting will follow the CONSORT rules.

A schematic diagram of the trial design, procedures and stages can be found in Supplement 2 of this Protocol.

4.3 A description of the measures taken to minimize/ avoid bias (randomization, blinding)

- Blinding of patients and treatment is not possible in the trial.
- Primary and most secondary endpoints are self-report measures. However, assessors will be scientific assistants from other parts of the respective trial sites who are not involved in the trial and blinded as to the treatment of the patient. As a check, they will be asked to guess the respective status of patient. Patients will be asked to withhold details of treatment from investigators and not to include intervention sessions in their health care utilisation interview..
- Potential participants will be screened consecutively in the co-operating somatic departments which are chosen for high base rates in patients with pain-dominant medically unexplained symptoms. All patients present in the respective unit for whom obvious exclusion criteria like severe organic disease or insufficient language ability do not apply will be informed about the study and asked to take part in an assessment of eligibility including the SOMS-7, the SF-36 and, if beyond cut-off, a diagnostic gold-standard interview (excerpt from SCID-1, adapted to test for criteria of MSD, see Henningsen et al. 2005).
- All therapy sessions will be audiotaped. Integrity of therapy will be monitored through regular supervision and independent rating of a random sample of audiotapes (2 sessions/ therapy) using the Sheffield Psychotherapy Rating Scale (Startup and Shapiro 1993). Therapists will fill out the adherence scale after every second session in order to prevent drift.
- Only therapists experienced in the use of psychodynamic-interpersonal psychotherapy will take part in the study. All therapists will be trained together in the application of the treatment manual in a central workshop before starting the treatment period; booster training sessions will aim at maintaining and further increasing therapist adherence.
- Patient´s use of medications and other therapies will be monitored. For additional treatment decisions during the intervention, patients will be referred to their General Practitioner

4.4 Description of the trial treatment and the dose of the intervention

Experimental Intervention
Manualized phase –defined 12 session intervention based on the principles of psychodynamic-interpersonal psychotherapy (Rudolf and Henningsen 2003, Margison 2002) applied to the purposes of patients with organically unexplained bodily symptoms (Guthrie et al. 1999)
Phase 1: Build-up of therapeutic relationship through symptom-oriented exploration and psychoeducation.
Phase 2: Broadening of the patient's explanatory model and symptom management.
Phase 3: Clarification of interpersonal symptom contexts and accompanying affects/ emotions.
Phase 4: Termination and transfer, including a detailed farewell letter.
The manualized intervention is adapted in Phase 1 to the leading symptom of the patient, otherwise it is identical across somatic specialities. The manual also defines the rules for taking part in parallel somatic treatments.
(For an extended English summary of the manual see Supplement 3a)

Control Intervention
Enhanced medical care for the respective symptom leading to current health care utilisation.

All patients are treated at specialized university departments, thereby assuring up to date treatment according to established guidelines. In addition to “treatment as usual” they will take part in three monthly protocol-defined case management counselling interviews aimed at increasing self care skills and shared decision making in illness behaviour. Patients assigned to ‘enhanced medical care’ will be informed of their research diagnosis and encouraged to inform their physician of their diagnosis, allowing them and their physicians to initiate primary care or speciality treatment without restrictions.
(for an English version of the guideline on “Enhanced Medical Care” see Supplement 3b).

Interpretation of differences between study arms concerning type of intervention and time spent with the patient
The design of the trial incorporates a confounding of type of intervention and time spent with the patient. Hence, this design does not control for unspecific effects of time spent with the patient. As a result, we will not be able to confirm the specificity of psychodynamic-interpersonal therapy for the treatment of patients with MSD.
This is acceptable because a) the main aim of the trial is a pragmatic one: is there anything effective that can help such patients as there is such limited evidence for effectiveness? If there is sufficient evidence that this treatment is beneficial, specificity can be addressed in later studies. b) To confirm specificity would involve a more elaborate and costly design, ideally a three limbed study PIT vs counselling vs. enhanced medical care. For one type of functional somatic syndrome, PIT has already been shown to be specific (Hamilton et al. 2000).

4.5 Expected duration of subject participation and description of the sequences and duration of all trial periods, including follow-up

The expected duration of subject participation is 16 months overall: A 4-week-period for screening, randomization and pre-therapy investigation, a 12-week-period for 12 weekly sessions of manualized psychodynamic-interpersonal psychotherapy or control intervention, final assessment after three months and follow-up-investigation 12 months after completion of therapy.
(For more details of trial schedule see trial diagram, Supplement 2)

4.6 Description of the „stopping rules“ or „discontinuation criteria“ for individual subjects, part of trial and entire trial

There are no predefined statistical stopping rules and no interim analyses in this trial.

Premature study termination can be due to the following reasons:
- Trial Steering or Data Monitoring and Safety Committee recommend to stop the trial
- Risk-benefit ratio is changed in relevant degree (e.g. through new external information)

A study site can be closed prematurely for the following reasons:
- Major protocol violations
- Inadequate patient recruitment
- Non-Compliance to the study requirements

For criteria for individual subject withdrawal see 5.3

4.7 Accountability procedures for the intervention, including the placebo(s) and comparator(s), if any

Does not apply in this trial.

4.8 Maintenance of trial treatment randomization codes and procedures for breaking codes.

Randomized allocation to one of two treatment arms after assessment of eligibility, and after obtaining informed consent from the patients for participation.
The patients selected for participation will be randomly assigned to either the control (enhanced medical care) or intervention group (manualized psychotherapy based on PIT principles) by a computer generated multi-block randomization schedule using the program “Rand”. This randomization schedule will be generated at the Coordination Centre for Clinical trials. The schedule will be a 1:1 ratio, permutated in setting blocks of 4, 6 and 8 patients. Randomization

will also be stratified by participating hospitals and settings. The concealment of treatment allocation is ascertained through central allocation in the KKS Düsseldorf by fax.
The trial is not blinded to patients or staff treating them as this is impossible with the intended trial design.

4.9 Identification of any data to be recorded directly on the CRFs (i.e. no prior written or electronic record of data), and to be considered to be source data.

The primary outcome and the majority of secondary outcomes are self report questionnaires that are considered to be part of the case report forms.

5 Selection and Withdrawal of Subjects

5.1 Subject inclusion criteria

- Patients screened positive and diagnosed in a structured interview in different somatic specialities with a diagnosis of pain-predominant multisomatoform disorder and a Quality of Life score as measured with SF-36 of 1 standard deviation or more below population norm.
- Signed informed consent

5.2 Subject exclusion criteria

- Age younger than 18 years
- Insufficient German language ability
- Insufficient cognitive abilities in the Mini Mental State (< 27)
- Severe and chronic somatic diseases (e.g. severe heart failure, encephalitis disseminata, dementia).
- Severe comorbid mental disorder causing major impairment of social functioning (e.g. schizophrenia, severe mood disorders, personality disorders, mental disorders due to substance abuse)

5.3 Subject withdrawal criteria (i.e. terminating intervention/ trial treatment) and procedures specification

a) When and how to withdraw subjects from the trial/ intervention

A patient can be withdrawn from the trial for the following reasons:
- Revocation of informed consent
- Loss to follow-up

A patient can be withdrawn from trial intervention for the following reason:
- If a patient needs acute medical treatment for proven somatic disease for the symptoms on the basis of which he was initially included.

Withdrawal of subjects from the trial will be done after consulting with the coordinating investigator. It is his responsibility to inform the Trial Steering and Data Safety and Monitoring Committee of this event immediately.

b) The type and timing of the data to be collected for withdrawn subjects

***a)* Whether and how subjects are to be replaced.**
Subjects will not be replaced during the trial. Drop out is taken into consideration in the sample size calculation.

b) **The follow-up for subjects withdrawn from interventional treatment/trial treatment**
- The follow-up for patients who are withdrawn from the interventional treatment will be identical to the follow-up of completers. These patients will be included in the intention-to-treat analysis.

6 Treatment of Subjects

6.1. The treatment(s) to be administered, including the dose(s), and the treatment period(s), including the follow-up period(s) for subjects for each intervention/trial treatment group/arm of the trial.

Experimental Intervention
Manualized phase –defined 12 session intervention based on the principles of psychodynamic-interpersonal psychotherapy (Rudolf and Henningsen 2003, Margison 2002) applied to the purposes of patients with organically unexplained bodily symptoms (Guthrie et al. 1999).

Control Intervention

Enhanced medical care for the respective symptom leading to current health care utilisation.
In order to increase motivation of patients in the control intervention, in addition to “treatment as usual” they will take part in three monthly manual-defined case management counselling interviews aimed at increasing self care skills and shared decision making in illness behaviour.

The treatment period will be 12 weeks/ 3 months in both limbs of the trial, the final assessment will take place after three months and follow-up at one year.

6.2. Medication(s)/treatment(s) permitted (including rescue medication) and not permitted before and /or during the trial.

Medication as prescribed by the General Practitioner and/ or the treating somatic physician(s) is permitted during the trial. Medication type and dosage will be monitored closely.

6.3 Procedures for monitoring subject compliance

Special ascertainment of non-compliance is not necessary in the planned trial as patient’s absence from therapy sessions is obvious and will be documented.

6.3. Procedures for monitoring treatment adherence by the therapist

All therapy sessions will be audio taped. Integrity of therapy will be monitored through regular supervision and independent rating of a random sample of audiotapes (2 sessions/ therapy) using the Sheffield Psychotherapy Rating Scale (Startup and Shapiro 1993). Therapists will fill out the adherence scale after every second session in order to prevent drift.
Furthermore only therapists experienced in the use of psychodynamic-interpersonal psychotherapy will take part in the study. All therapists will be trained together in the application of the treatment manual in a central workshop before starting the treatment period; booster training sessions will aim at maintaining and further increasing therapist adherence.

7. Assessment of Efficacy

7.1. Specification of the efficacy parameters and justification for their clinical relevance

Efficacy of the intervention will be determined through the primary and secondary endpoints of the trial as described in 4.1.
Treatment effects in patients with multisomatoform disorder, if present, regularly show up primarily in a lesser degree of impairment by the symptoms rather than in a disappearance of the symptoms – in keeping with the notion that somatoform disorders are “disability syndromes”. Therefore it is justified to use a health related quality of life measure as primary endpoint. The secondary endpoints all assess important aspects either of psychopathology or of potential mediators of change. Information about validation of the measures is given above.

7.2. Methods and timing for assessing, recording, and analysing of efficacy parameters

Efficacy parameters will be assessed before and after the treatment period and after one year. Most efficacy parameters are self-report data. In addition, there will be an assessment of psychophysiological measurements at three month.

8. Assessment of Safety

8.1. Specification of safety parameters.

All complications and unanticipated adverse events will be reviewed by the coordinating investigator. The coordinating investigator is responsible for identifying issues that warrant immediate action and the evaluation of trends of consequence.

For the purposes of this study we will define adverse and serious adverse events according to the ICH-GCP guidelines (guidelines for Good Clinical Practice; ICH-Harmonised Tripartite Guideline; EMEA Status as of June 1996) as follows:

Adverse Event (AE): Any untoward medical occurrence in a patient or clinical investigation subject which does not necessarily have a causal relationship with this treatment. An adverse event (AE) can therefore be any unfavourable and unintended sign (including an

abnormal laboratory finding), symptom, or disease temporarily associated with the treatment investigated in the study, whether or not related to the treatment product.

In PISO, AEs are likely to occur with medium to high frequency, as the population studied is defined by frequent and frequently changing somatic symptom complaints. It is up to the investigator at the respective trial site to decide whether complaints of the patients are to be seen as anticipated in this sense or whether they are judged as being unanticipated and hence necessitate immediate information of the coordinating investigator.

Serious adverse event (SAE): Any untoward medical occurrence that:

- results in death,
- is life-threatening,
- requires inpatient hospitalisation or prolongation of existing hospitalisation,
- results in persistent or significant disability/incapacity
- congenital anomaly or birth defect.

In PISO, increased depressivity and/ or suicidality is considered as SAE and should be reported in the same way.

8.2. Methods and timing for assessing, recording and analysing safety parameters

Assessment of bodily and other complaints as well as of depressivity and suicidality is an integral part of the 12 treatment sessions in the experimental intervention and of the 3 case management sessions in the control condition within PISO.
Each treatment session will be audiotaped, in addition, written notes of the relevant aspects of the session will be kept by the therapist.
All safety parameters will also be recorded at final assessment at 3 months and follow-up at 1 year.

8.3. Procedures for eliciting reports of and for recording and reporting adverse event and intercurrent illnesses

All adverse events, including intercurrent illnesses and an increase in severity or frequency of a sign/symptom of a concomitant disease will be reported on and documented.
Unanticipated adverse events are to be documented on an "Adverse Event (AE) / Serious Adverse Event (SAE)" page of the case report form. All findings in patients experiencing unanticipated adverse events must be reported there and in the patient's medical records.
The investigator should state whether the event is considered as serious, if a causal relationship to trial therapy exists, and note the severity of the event.
The severity of adverse events has to be classified as:

- mild = normal activities of daily life can be performed
- moderate = normal activities of daily life are restricted
  severe = normal activities of daily life cannot be performed

For all SAEs - independent of a possible causal relationship - the investigator will submit a SAE report to the recipient (coordinating investigator) indicated on the cover page of the form **within 24 hours** after having gained knowledge of the event. The investigator is obliged to provide a complete documentation of the course and the measures taken, if necessary with the submission of original findings.
In addition, any SAE will be registered in the "Adverse Event Form" of the CRF.

After receipt of the initial report, the coordinating investigator will review the information and contact the investigator if necessary, to obtain further information for assessment of the event.

The investigator will submit, if necessary, copies of all these reports to the independent ethics committee concerned.

8.4. Type and duration of the follow-up of subjects after adverse events

If required, a follow-up report including all new information obtained on the serious adverse event will be prepared and sent to the address of the SAE-form recipient or it will be collected by the monitor. The report should be marked "Follow-up report". If a SAE makes it necessary to initiate inpatient treatment, i.e. on a psychiatric ward due to acute suicidality, discharge information will be collected and treated as a further follow-up report.

9. Statistics

9.1. Description of the statistical methods to be employed

The data will be listed per center, per setting and per patient. Descriptive statistics will be calculated for each treatment group. Demographic and baseline data will be compared using appropriate statistical techniques.

For confirmatory analysis a modeling approach will be applied. The null hypothesis tested is: no treatment difference between control and intervention group with respect to the primary endpoint. A linear model with mixed effect will be used:

$$y_{i,j,c,s(c)} = \mu + \alpha_j + \eta_c + \zeta_{c,s(c)} + \varepsilon_{i,j,c,s(c)},$$

where $y_{i,j,c,s(c)}$ is the response of individual *i* (*i=1,...,$m_{c,s}$*) randomize to treatment *j* (*j=1, 2*) for center *c* (*c= 1, ...,C*) and setting *s(c)* ($s_{(c)}$ *= 1, ..., n_c*). The notation indicates two treatment assign, and two level of stratification (patients within settings and settings within centers) with number of settings varying within centers. The total number of observations per treatment group is $\sum_c \sum_s m_{c,s}$.

The fixed-effects parameters in the model are μ (the grand mean) and α (the group effects). The random effect η indicates center effect, ζ setting effect and ε measurement error. These three random variables will be assume statistically independent each other, with mean zero and component of variances τ_η, τ_ζ , and τ_ε respectively.

The parameters of interest in this analysis are the mean of the control group $\mu+\alpha_1$ and the contrast of means α_1-α_2 (which indicates treatment effect).
Estimation of model parameters will be calculated with REML (restricted maximum likelihood) method. Treatment effect will be tested with a likelihood ratio test (LRT at the alpha-level of 0.05). Additionally, centers and settings effect will be predicted with the best linear unbiased predictors (BLUPs) of the random effects. Asymptotic confidence intervals will be reported for all parameters in the model.

Distributional assumptions will be assessed (e.g. residual analysis) and transformation on the response variable will be done if it is necessary. Additionally, if adjustments for covariates are necessary they will be included in the model.

9.2. The number of subjects planned to be enrolled

Sample size calculation is motivated by the primary endpoint, based on the Physical Component Summary of SF-36 (PCS). We assessed the PCS for somatoform disorders in the general population with a mean value of 45,76 (SD=10.03) (Schmitz and Kruse, German National Health Interview and Examination Survey - Mental Health Supplement (GHS-MHS) 1998, public use file) and, clinically more realistically, with 37 (SD=12.5) in a sample of 186 patients in the University Hospital Heidelberg, treated in the outpatient departments for psychosomatics and orthopaedic chronic pain, comparable to the trial criteria (Henningsen et al. 2005).
In the trial an effect size of 0.5 is assumed, which is based on the following considerations: Clinically important change on the SF-36 PCS (ie what patients regard as important change) has been considered as follows: small change= 2.5; moderate = 4.5 and large = 6.5 (Wiebe et al 2002). In a previous trial of PIT in a functional somatic disorder (Creed et al. 2003), patients who received PIT improved by a mean of 5.5 points on the PCS compared to those receiving usual care, and this was associated with a reduction of median number of days of restricted activity per month from 5 to 0. Therefore, assuming an effect size of 0.50 on the basis of a standard deviation of 12.5 implies a clinically significant, large change of 6.25 in this measure of physical quality of life.

A sample size of 63 in each group will have 80% power to detect an effect size of 0.50 assuming a mean value of 37 in the control group and a known common standard deviation of 12.5 using a two group normal test with a 0.05 two-sided significance level.

This sample size calculation is based on the standard statistic

$$n = 2\sigma^2 / \theta^2 (z_{0.8} - z_{0.025})^2 \approx 63,$$

where $\sigma^2 = 12.5^2$, $\theta = 6.25$, $z_{0.8} = 0.84$, $z_{0.025} = -1.96$ and effect size is θ/σ.

Several scenarios are considered for the number of patients per group. Table 1 shows a deterministic sensitivity analysis for different values of effect size. On the bases of the above requirements the sample size of n = 63 per group was determined. Although, the sample size is very sensible to possible changes of effect size.

Table 1. Sample size estimation per group for different effect size and power ($\alpha = 0.05$, 2-sided).

effect size	*power 70%*	*power 80%*	*power 90%*
0.6	34	43	58
0.55	40	52	70
0.50	49	**63**	84
0.45	61	78	103
0.40	77	98	131

In order to assess if the trial could be underpowered, a further probabilistic sensitivity analysis on the sample size and the power is carried out. This analysis puts uncertainty over the parameters θ and σ^2. It is assumed that the prior mean of θ is 6.25 with standard deviation 1, while for σ is assumed a prior mean of 12.5 and standard deviation of 1 (subject to the constraint of σ to be positive).

A Monte Carlo simulation method is applied to simulate values of θ and σ^2 from their prior distributions, substituting them into the sample-size formula and the resulted simulation values n_1^* ,..., n_B^* recorded.

For a fixed *n*, the power is given by

$$power = \Phi(\sqrt{(n\theta/2\sigma^2)} + z_{0.025}).$$

If is decided to use 63 patients per group, values for the *power* can be simulated using the same methodology, resulting in simulated values $power_1^*$... $power_B^*$.

A total of B=10000 simulated values n_b^* and $power_b^*$ were carried out. Figure 1 gives the density estimation of these two quantities. They present a clear uncertainty for sample size and power values.

To achieve a fixed power of 80 % the median of the simulated sample size n^* is 88. For a fixed n=63 the median of the simulated $power^*$ values is 65%. These results indicates that the deterministic sample size calculation may give optimistic results and a larger sample size should be used to achieve a power of 80%.

Fixing n=88 in the power formula and simulating again B=10000 gives a median power of 79.6 %, which is more close to the trial power requirements. The recommended sample size for this trial is n=88 per group.

Figure 1. *Left panel:* distribution for necessary sample size n to achieve 80% power. *Right panel:* power distribution with a fixed n = 63 patients per group.

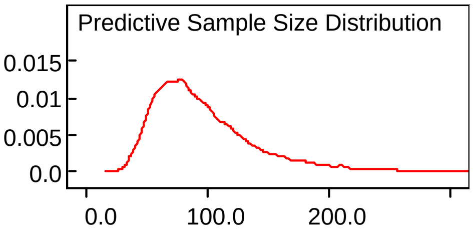

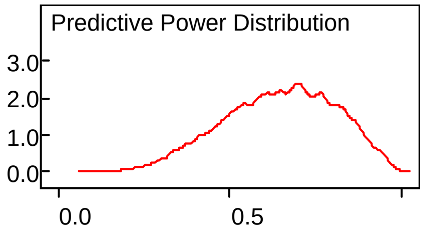

9.3. A demonstration of the potential for recruiting the required number of suitable subjects within the intended period

A pilot study was performed in all trial centres in November and/ or December 2004. All consecutive patients in the collaborating somatic departments were pre-screened and patients without evident reasons for exclusion (major organic disease, age) were asked for their informed consent to take part in the pilot assessment and interview. Variations in rates of pre-screening exclusions are mostly due to different percentages of organically explained disorders in the different settings. Patients were given the SOMS-7 (see 5.9) and those above cut-off took part in an excerpt of the SCID-Interview to test for for presence of Multisomatoform Disorder (MSD) and were checked for other inclusion and exclusion criteria as defined in 5.6. Eligible patients were informed about the planned study orally and with written material, afterwards they were asked whether they would agree to take part in this study if it became available.

**Trial Centre**	**Weeks of screening**	**Number of assessed out of total patients seen (N)**	**Eligible patients w. MSD (N)**	**Patients agreeing to take part (N)**
Heidelberg[1]	3	90 out of 120	17	14 (19 per month)
Düsseldorf[1,4]	3	38 out of 52	8	6 (9 per month)
Hannover[1]	2	10 out of 25	6	6 (12 per month)
München[1]	2	20 out of 32	6	4 (8 per month)
Münster[2,3]	3	11 out of 168	6	6 (8 per month)
Regensburg[2,3]	4	26 out of 200	6	6 (6 per month)

Setting: [1]Pain Centre; [2]Internal Medicine; [3]Neurology; [4]Dental Medicine

Although the rates of eligible patients agreeing to take part are different between the centres, the results of this pilot study confirm that each centre will be able to fulfil the recruitment rate of approx. 4 eligible patients per centre per month agreeing to be randomized into the study.

9.4. Criteria for the termination of the trial

There are no formal statistical stopping rules defined for the trial. Premature study termination can be due to the following reasons:
- risk/ benefit ratio is changed
- DMSC recommends to stop the the trial

Regular study termination will take place when the calculated numbers of patients in both arms have completed their follow-up.

9.5. *Procedure for accounting for missing, unused, and spurious data according manuals*

Missing data in the primary endpoint will be handled according to the manual of the SF-36.

9.6. *Procedures for reporting any deviation(s) from the original statistical plan*

Any deviations from the original statistical plan will be described and justified in protocol and in the final report, as appropriate.

9.7. **Selection of subjects to be included in the analyses (e.g. intention-to-treat, all randomized subjects, all eligible subjects, evaluable subjects.)**

The study population will be as follows:

- Intention- to- treat- population
  This population consists of all subjects randomised, irrespective of whether they received the

experimental or control intervention. Subjects will be analysed according to the intervention to which they have been randomised.

- Per- protocol population
  This population is the subset of all subjects included in the trial which excludes those subjects who had a major protocol deviation.

The efficacy analysis with primary and secondary endpoints criteria will be done in the intention-to- treat population.
In addition, for informative purposes, a less conservative analysis based on the per- protocol-population will be done for the primary and secondary endpoints.
Conclusions of the trial will be based only on the intention- to- treat population.

10. Direct Access to Source Data/Documents

We ensure that it will be specified in written agreements that the investigators institutions will permit trial-related monitoring, audits, IRB/IEC review, and regulatory inspections, providing direct access to source date / documents.

11. Quality Control and Quality Assurance

All efficacy parameters are derived from standardized and validated instruments.

All measures concerning monitoring/quality assurance will be conducted in accordance to ICH-GCP. Monitors are to be qualified by education, training, and experience.

11.1. Initiation Visit

The monitor will give the investigator an extensive introduction to study protocol and study requirements. He/she is responsible for ensuring and documenting that each investigator clearly understands and accepts the responsibilities and obligations incurred in conducting a clinical study, for example how to obtain informed consent in accordance with GCP and the way of documenting adverse events.

11.2. Periodic Monitoring Visits

The monitor will visit each site regularly. The number of visits is dependant on the number of subjects enrolled and the scope of the contractual obligations at each site. A maximum of 8 visits per site including initiation and termination visit is anticipated.

The following key variables will to be monitored to 100%:
- Inclusion criteria
- Exclusion criteria
- Informed consent
- Primary and secondary outcome variables

The monitoring of other parameters will be randomly specified.

Monitoring will be conducted according to the harmonized Standard Operating Procedures (SOPs) of the KKS-AG, study specific processes will be described in detail by working procedures.

Monitoring reports will be prepared for all visits. Reports must always include the following information:

- Date of the visit;
- List of study site personnel present;
- State of enrolment
- A summary of the findings, problems, and actions taken to correct any deficiencies.

11.3. Study termination visit

All routine monitoring functions must be performed prior to the study termination visit; all queries generated by the data management should be answered. The monitor proves all study site documents to be complete and correct. He/she reminds the investigator of the obligation to retain the records according to regulatory requirements.

12. Ethics

As it is based on common therapeutic strategies in patients with medically unexplained symptoms, the intervention in PISO is not experimental. Psychotherapeutic interventions like this have repeatedly been shown to be relatively safe, but all have potential side effects. In particular, there is a risk that during the intervention patients may transiently become more depressive and/ or suicidal as a sign of altered emotional processing of life stresses. Close monitoring of this risk by the therapist is assured through the treatment manual and is helped by the fact that the intervention is taking place not in group, but in single patient format. Appropriate action (e.g. antidepressive medication, psychiatric in-patient referral) is defined in a manual for clinical care that will be adapted to the local situation of each centre. This manual also defines the clinical care for drop outs.
The procedures protecting against bias and defining termination of the study, study site closure and patient withdrawal are laid down in the Trial Protocol; they also contribute to patients' safety.
Before deciding to take part in the trial, patients´ informed consent will be obtained and legal and regulations of the ethics committees involved will be followed to assure patients' safety and confidentiality.
For the pilot trial assessing achievability of recruitment rate, the patient information and consent form for the study itself (and for the pilot trial) were checked and agreed upon by the ethics committee of the University Hospital Düsseldorf on Nov. 30th, 2004

12.1. Independent ethics committee

The Coordinating Investigator will submit the final protocol and proposed Patient Information and Informed Consent documents to the Independent Ethics Committee (IEC), which is responsible for the Principal Investigators site. Copies of all these documents will be given to all Ethics Committees, which are responsible for other sites, participating in the study. The clinical trial will not be initiated until appropriate documentation of IEC approval of the study protocol and the Patient Information/Informed Consent has been received by all participating sites.

Modifications to the protocol will be submitted to the IEC for approval.

Potential modifications will be implemented only after IEC written approval of these changes has been received by the participating sites.

The Investigators will make appropriate and timely reports to the IEC as required by applicable government regulations and IEC policy. In addition to the progress reports, all known information regarding serious adverse events (SAE), whether observed at their clinical site or at another site participating in the clinical study, will be reported to the IEC.
It is the Investigators' obligation to provide the Coordinating Investigator with copies of all study-related correspondence with the IEC in a timely fashion and to retain original within an appropriate filing system.

12.2. Ethical conduct of the study

12.2.1 Regulatory requirements, guidelines and standards

The trial will be performed according to ICH-GCP. Good Clinical Practice (GCP) is an international ethical and scientific quality standard for designing, conducting, recording and reporting trials that involve the participation of human subjects. Compliance with this standard provides public assurance that the rights, safety and well-being of trial subjects are protected,

consistent with the principles that have their origin in the Declaration of Helsinki, and that the clinical data are credible.

Declaration of Helsinki
The conduct of the trial is going to be in accordance with the declaration of Helsinki (2000, Edinburgh, Scotland).

12.2.2 Authority approvals and registration

Requirements of Health Authorities and national regulations will be respected. The trial will be registered immediately in receipt of the notification of award by the BMBF/DFG at http:www.controlled-trials.com

12.3. Patient Information and Informed Consent

Patient Information and Consent forms are to be approved by the responsible ethics committees and recruitment cannot commence before approval has been given.

Written informed consent must be obtained from all patients prior to the study inclusion. The patient and the investigator will sign the current IEC-approved version of the consent form and a signed copy of the consent form will be given to the patient. The data that consent was obtained will be recorded in the patient's medical record.

Original, signed consent forms must be made available to the study monitor when requested.

The potential risks and benefits of participation in this study are clearly identified in the patient consent form. Any patient eligible for participation in the investigation according to the inclusion will be advised of these potential risks and benefits before they sign the patient consent form and enrol in the study. Every patient has the right to refuse to participate in this investigation or to withdraw from the investigation at any time without any negative consequence.

A draft of the patient information and consent form is provided in Supplement 4.

13 Data Handling and Record Keeping

The data management will follow a Remote Data Entry approach. The electronical Case Report Form (eCRF) will be implemented in a modern Clinical Data Management System (CDMS) with Electronical Data Capture functionality (EDC). The system complies with the relevant international standards and provides the capability to perform the major data management activities within a consistent, auditable and integrated electronical environment (query management, data entry, data validation).

The data will be collected primarily on paper CRFs, which will be transcribed to the eCRF by the sites personnel (investigator or assistant personnel). The transcription of data in any case will have to be signed by the investigator. Most of the data validation will be performed by the system at data entry (data type, format, univariate and multivariate validation checks). The monitor will regularly review the eCRFs for preparation of her/his on site visits and identify visually incomplete or inconsistent data. More complex data validation will be done by programming validation checks on the data back end (Oracle database). So the data cleaning process will be active from the beginning, to provide a good data basis for data monitoring and the trial progress monitoring, reducing significantly the number of queries to the sites. Queries can be manually raised by the monitor or by the data manager or automatically by the system.

The query management is performed electronically. The collected data that are transferred to the coordinating centre will only include pseudonymized data. The connection is secured by SSL-technology. Archiving of the clinical database including the audit trail can be provided by the coordinating centre in a machine independent format. Sites can be provided with an electronical CRF of their patients if necessary at study termination. After database lock data can be immediately imported into SAS statistical software systems (or other systems like SPSS) for analysis.

14 Insurance

Where appropriate and in accordance with the national regulations a patient insurance is provided for the patients taking part in the trial.
Insurance cover for transport accidents will be provided where appropriate.

15 Publication Policy

Results of the study will be published in international peer-reviewed journals aiming at a readership in psychosomatic medicine and psychiatry as well as in the somatic specialities involved (pain medicine, neurology, internal medicine etc.). As member of the editorial board of “Psychosomatic Medicine”, the coordinating investigator has good knowledge of the requirements for successful publications.
In addition, a scientific meeting will be organised to promote the results and increase the acceptance of psychosomatic treatments among professionals. A study-specific homepage will facilitate information about the trial and its results also among patient groups and other interested parties.
The experiences with and results of the study will also be used to revise the guidelines on diagnosis and treatment and to promote the distribution of the manualized psychotherapeutic intervention for training of doctors and for continuing medical education, especially in so-called Psychosomatic Basic Care Programs, which doctors have to take part in order to get their registration as somatic specialist. In addition, pre-existing contacts to public institutions specializing on the reporting of medico-scientific progress like television programmes will be used to raise public awareness for the possibilities of such a psychotherapeutic intervention and for the risks of continued somatic diagnosis and treatment in this group of patients

16 Supplements

Supplement 1: Investigators and Trial Sites

Trial Site Heidelberg

(Coordinating Investigator / Investigator
Peter Henningsen, Priv.Doz. Dr.med.
Deputy Head/ "Leitender Oberarzt"; DoB 11.02.1959, German
University Hospital Heidelberg, Dept. of Psychosomatic Medicine
Tel. +49-6221-56-5865; Fax -33586, e-mail: peter.henningsen@med.uni-heidelberg.de
Private address: Wilhelmstr. 9, 69115 Heidelberg, Tel:+49-6221-184900
- until end of August 2005)

Investigator
Nina Sauer, Dr.med.
Oberärztin; D.o.B. 21.07.1967, German
University Hospital Heidelberg, Dept. of Psychosomatic Medicine
Thibautstr. 2, 69115 Heidelberg, Germany
Tel: +49-6221-56-5865; Fax: -33586, e-mail: nina.sauer@med.uni-heidelberg.de
Private address: Schulzengasse 2, 69120 Heidelberg

Co-Investigator
Bernd Loewe, Priv. Doz. Dr. med., Dipl.-Psych.
Oberarzt; DoB 01.01.1966, German
Department of Psychosomatic Medicine, University of Heidelberg
Im Neuenheimer Feld 410, 69120 Heidelberg, Germany
Tel: +49-6221-56-38738; Fax: -5749, e-mail: bernd.loewe@med.uni-heidelberg.de
Private address: Parkstrasse 11, 69126 Heidelberg

Principle physician from cooperating units
Prof. Dr. H. Bardenheuer
Head of Center
Center for pain management
Department of Anaesthesiology
University Hospital Heidelberg

Trial Site Düsseldorf

Investigator
Johannes Kruse, Priv.Doz. Dr.med.
Deputy Head / "Leitender Oberarzt"; D.o.B. 15.01.1959, German
Department of Psychosomatic Medicine, University of Düsseldorf
Bergische Landstrasse 2 , 40629 Düsseldorf, Germany
Tel: +49-211-922-4702, Fax: -4707, e-mail: kruse@uni-duesseldorf.de
Private address: Stadtparkinsel 22; 41515 Grevenbroich

Principle physician from cooperating unit
Alfons Hugger, Priv. Doz. Dr.
Associate Professor
Department of Prosthodontics, Westdeutsche Kieferklinik,
University Hospital of Düsseldorf
Moorenstr. 5, 40225 Düsseldorf, Germany

Trial Site Hannover

Investigator

Martin Sack, Dr.med.
Oberarzt; D.o.B. 13.06.1961, German
Dept. of Psychosomatic Medicine and Psychotherapy, Hannover Medical School
Carl-Neuberg-Str. 1, 30625 Hannover, Germany
Tel: +49-511-532-3195, Fax: -3190, e-mail: sack.martin@mh-hannover.de
Private address: Kirchstr. 12, 30449 Hannover

Principle physician from cooperating unit
Priv.-Doz. Dr.med. Matthias Karst
Dept. of Anaesthesiology, Pain Clinic (Schmerzambulanz)
Hannover Medical School (Medizinische Hochschule Hannover)
Carl-Neuberg-Str. 1, 30625 Hannover

Trial Site München

From September 2005:
Coordinating Investigator
Peter Henningsen, Prof. Dr. med.
Head of Dept./ D.o.B. 11.02.1959, German
Dept. of Psychosomatic Medicine, Psychotherapy and Psychological Medicine
Langerstr. 3, 81675 München, Germany
Tel: +49-89-4140-4310, Fax: -4845, e-mail: p.henningsen@tum.de
Private address: Franz-Joseph-Str. 18, 80801 München

Investigator
Harald Gündel, Priv. Doz. Dr.med.
Oberarzt, D.o.B. 16.05.1963, German
Dept. of Psychosomatic Medicine, Psychotherapy and Psychological Medicine
Langerstr. 3, 81675 München, Germany
Tel: +49-89-4140-4316, Fax: -4845, e-mail: h.guendel@lrz.tu-muenchen.de
Private address: Schumannstraße 12, 81679 München

Principle physician from cooperating unit
Prof. Dr.med. Dr.rer.nat Thomas R. Tölle
Deputy Head, “Geschäftsführender Oberarzt“
Head, Interdisciplinary Pain Treatment Centre
Dept. of Neurology, Technische Universität München
Möhlstraße 28, 81675 München

Trial Site Münster

Investigator
Gudrun Schneider; Priv. Doz. Dr.med.
Deputy Head/ “Leitende Oberärztin“; D.o.B. 21.05.1964, German
Department of Psychosomatic Medicine, University Hospital of Münster
Domagkstr. 22, 41849 Münster, Germany
Tel: +49-251-835-2910, Fax: -2903, e-mail: schneig@mednet.uni-muenster.de
Private address: Uppenbergstr. 12, 48149 Münster

Co-Investigator
Markus Burgmer, Dr.med.
Scientific Assistent, D.o.B. 17.06.1968, German
Department of Psychosomatic Medicine, University Hospital of Münster
Domagkstr. 22, 41849 Münster, Germany
Tel: +49-251-835-2910, Fax:-2903, e-mail: Markus.Burgmer@mednet.uni-muenster.de
Private address: Augustastr. 13, 48153 Münster

Principle physicians from cooperating units

Prof. Dr. E.B. Ringelstein
Prof. Dr. I. W. Husstedt
Department of Neurology, University of Münster

Prof. Dr. Dr.h.c. W. Domschke
Department of Internal Medicine B
University of Münster

Trial Site Regensburg

Investigator
Claas Lahmann, Dr.med.
Scientific Assistant; D.o.B. 19.08.1975, German
Psychosomatic Unit, University Hospital Regensburg
Franz-Josef-Strauss-Allee 11, 93053 Regensburg, Germany
Tel: +49-941-944-7243; Fax: -7377, e-mail: claas.lahmann@klinik.uni-regensburg.de
Private address: Taubengässchen 5, 93047 Regensburg

Co-Investigator
Thomas Loew, Prof. Dr.med.
Head of Dept.; D.o.B. 28.03.1961, German
Psychosomatic Unit, University Hospital Regensburg,
Franz-Josef-Strauss-Allee 11, 93053 Regensburg, Germany
Tel: +49-941-944-7241; Fax: -7377, e-mail: thomas.loew@klinik.uni-regensburg.de
Private address: Schlüsselackerstrasse 10, 93161 Sinzing

Principle physicians from cooperating units

Günther Riegger, Prof. Dr. med.
Clinical Director
Department of Internal Medicine II
University Hospital Regensburg
Franz-Josef-Strauss-Allee 11, 93053 Regensburg, Germany

Jürgen Schölmerich, Prof. Dr. med.
Clinical Director
Department of Internal Medicine I
University Hospital Regensburg
Franz-Josef-Strauss-Allee 11, 93053 Regensburg, Germany

Ulrich Bogdahn, Prof. Dr. med.
Clinical Director
Department of Neurology
University Hospital Regensburg
Universitätsstrasse 81, 93053 Regensburg, Germany

Michael Pawlik, Dr. med.
Deputy Head of pain clinic
Dept. of Anaesthesia
University Hospital Regensburg
Franz-Josef-Strauss-Allee 11, 93053 Regensburg, Germany

Trial Consultant
Elspeth Guthrie, Prof. Dr.
Professor of Psychological Medicine, D.o.B. 22.02.1958, British
Department of Psychiatry, University of Manchester
Rawnsley Building, CMHT, Oxford Road, Manchester M13 9WL
Tel: +44-161-276-5383, Fax:- 273 2135, e-mail: Elspeth.A.Guthrie@man.ac.uk

Private address: The White House, Rowarth, High Peak, Derbyshire, SK22 1EB, UK

Biostatistics / Monitoring / Data Management

Principle Biostatistician
Christian Ohmann, Prof. Dr. rer.nat.
Head of Dept., D.o.B. 6.3.1949, German
Coordination Centre for Clinical Trials
University of Düsseldorf
Moorenstr. 5, 40225 Düsseldorf, Germany
Tel: +49-211-811-9700; Fax:-9702, e-mail: ohmannch@uni-duesseldorf.de
Private Adress: Prinz-Georg-Str. 51, 40477 Düsseldorf

Second Biostatistician
Pablo Verde, Master of Science in Biostatistics
Scientific Assistant
Coordination Centre for Clinical Trials
Heinrich-Heine-Universität Düsseldorf
Moorenstr. 5, 40225 Düsseldorf, Germany

Supplement 2: Trial diagram

Months 1-2: Preparation of trial
Meetings of investigators for arranging details with the trial sites

↓

Months 3-5: Training of therapists
Only therapists experienced in the use of psychodynamic-interpersonal psychotherapy will take part in the study. All therapists will be trained together in the application of the treatment manual in a central workshop before starting the treatment period

↓

Months 5-16: Screening
Potential participants will be screened in co-operating somatic depart-ments. All patients present in respective unit for whom obvious exclusion criteria do not apply will be informed about the study and asked to take part in two part screening.
1st part: first screening for somatoform disorders by screening questionnaire
2nd part: if appropriate detailed screening with questionnaires, interview;
patient information and informed consent

↓

Randomisation
Randomization schedule will be generated at the Coordination Centre for Clinical Trials

↓

Pre-Therapy Investigation
questionnaires, heart-rate-variability-testing

Months 6-19: Intervention

Experimental Intervention (12 weeks)	Control Intervention (12 weeks)
Manualized phase–defined 12 session intervention based on the principles of psychodynamic-interpersonal psychotherapy applied to the purposes of patients with organically unexplained bodily symptoms.<br>Phase 1: Build-up of therapeutic relation-ship through symptom-oriented exploration and psychoeducation.<br>Phase 2: Broadening of the patient`s ex-planatory model and symptom manage-ment.<br>Phase 3: Clarification of interpersonal symptom contexts and accompanying affects/ emotions.<br>Phase 4: Termination and transfer, including a detailed farewell letter.	Enhanced medical care for the respective symptom leading to current health care utilisation.<br>All patients are treated at specialized university departments, thereby assuring up to date treatment according to established guidelines. In order to increase motivation of patients in the control intervention, in addition to “treatment as usual” they will take part in three monthly protocol-defined case management counselling interviews aimed at increasing self care skills and shared decision making in illness behaviour.

Months 9-22: Final assessment at 3 months
Investigation of outcome-measures: questionnaires, interview, heart-rate-variability-testing

↓

Months 20-31: Follow-Up at 1 year
Investigation of outcome-measures: questionnaires, interview

Supplement 3a: Manual of experimental intervention

Psychodynamic-interpersonal treatment of patients with multisomatoform disorder within "PISO"

Treatment manual – abbreviated English translation

Peter Henningsen, Johannes Kruse, Gudrun Schneider, Markus Burgmer, Nina Sauer, Claas Lahmann; Thomas Loew, Martin Sack, Harald Gündel

Within the scope of the PISO-Study, the effect of a 12 session, phase-defined and guideline-based intervention will be evaluated. This intervention will be offered to patients suffering from a multisomatoform syndrome in different contexts of somatic medicine (criteria according to Kroenke et al. 1997). Patients with hypochondriasis are not included in the study. In what follows, the principles and structure of the intervention will be presented.

General note about the manual:

The psychosomatic intervention is based on the principles of psychodynamic-interpersonal therapy applied to patients with bodily complaints as elaborated in Germany by Rudolf and co-workers and in the UK by Hobson, Guthrie and co-workers.
The set up of the manual is regarded as a „phase-adapted columns model": The relevant basic topics ("columns", e.g. psychoeducation, experience of symptoms/ body or relationships) represent the background in all therapy sessions. Phases are defined by the prominence of certain topics, they assume center stage in the therapeutic dialogue.
Before the beginning of the therapy, patients are to be informed about the manualized character of the therapy.
After every session, therapist and patient discuss a brief feedback and summary about the session topics in order to enhance transparency and to mark out therapeutic progress and improve the motivation for therapy.
A permanent part of the interventions is the body-orientated work using relaxation elements which start in the first or second therapy session depending on the individual situation. For this purpose the Progressive Muscle Relaxation technique (PMR) is introduced (imaginative techniques are an alternative).

Four general psychotherapeutic rules

The initial phase of forming the relationship and the helping alliance is of particular importance in the intervention. The following four general rules regarding psychotherapeutic techniques are applied especially in the initial phase of the intervention; considering the total duration of 12 sessions only these rules remain important for the psychotherapeutic orientation in the further phases of the therapy as well.

1. **Accepting the symptom complaint**

Bodily complaints and the relational experiences and episodes that are related to them are the primary material of the therapy not only in the first session, i.e. they are in no way a waste of time or „resistance". In this context the therapist is not only passive and open for the complaints, but he/she also organises and structures the complaint by giving comments and showing reactions. In this way the patient can learn to see the complaints– on the grounds of an increasingly stable therapeutic helping alliance – more and more as being influenced by a context of relational experiences and emotions (compare below, phase 1 of intervention).

2. **Tangential conduct of therapeutic dialogue**

This kind of conducting a conversation is derived from the therapeutic attitude which is described above. The actively supporting approach means basically that it gives and does not demand something, e.g. gives own statements, instead of asking questions most of the time or demanding psychological understanding or answers. If psychological topics are introduced, then rather casually, using general terms like „stress“, „strain“ or similar terms, and rather suggesting explanations from one's own experiences than telling the patient that this applies for him („From my own experience I know the situation that...“) – motto: „Psychotherapeutic processes come about most easily when they are not explicitly targeted on at the beginning, neither by framework nor by using special terms“.

3. **No early confrontation of the patient with "resistance" or with psychological interpretations**

This rule is logically derived from the above mentioned. Especially at the beginning, interpretations of possibly existing psychosocial correlations of the (bodily) complaints, however obvious they might be to the therapist, can be easily misunderstood by the patient, as if the therapist does not really believe in the existence and/ or seriousness of the complaints. That might not be a helpful motivation for reflexion of cognitions and emotions.

Also it makes sense not to enter into a discussion about the causes of somatic complaints, especially not in an „either-or“ fashion of "organic or psychogenic". It is important to accept the possibility of organic causes for the complaints, but at the same time it should be pointed out that an explanation of exclusively organic causes is not sufficient to understand the complaints sufficiently (i.e. an attitude of „as well as“).

The patient is not confronted with problematic and subconscious aspects of his behaviour and experience because such a confrontation could have a negative effect on the helping alliance (exception: acute threat to self and others). In an atmosphere of respect and acceptance the patient's statements are understood as his/her special form of communication and as a resource to enter into a communicative process with the conversation partner.

4. **Reacting in a selectively authentical way**

In the statements about the therapeutic attitude and the rules so far mentioned, the technique of „reacting in a selectively authentical way“ is already implicitly contained. This „principle of answering“ instead of a „principle of interpretation“ has been shown to be reliable in the psychotherapy with patients suffering from impairments of personality structure; e.g. in the so-called psychoanalytic-interactional psychotherapy where it is the most important form of intervention. In the interaction with patients with somatoform disorders this principle is also helpful and necessary for creating the helping alliance, regardless of the level of personality structure. This also means that the therapist shows an actively sympathetic and accepting, not neutral attitude towards the patient.

Phases and Elements of the Intervention

The definition of the phase a certain session belongs to is derived from the specific contents of this phase that usually is followed over a longer period in the session. Besides this, the session usually also contains elements of previous phases (see „adapted column model“).

Therapy Phase 1

**Symptom understanding**	
Topic	History taking, psychoeducation, relaxation
Time	3-4 sessions, 1st hour > 60 min, optional two double hours
Agreements	Explanation of the structure and content of the psychosomatic intervention<br>Explanation of the setting (location, appointment, what to do when the appointment has to be postponed, pointing out that it is necessary to participate regularly, agreement that it is possible to cancel two appointments at most that can be made up for)<br>Recommendation not to see other somatic therapists during the therapy if possible, at most the family doctor, agreement to report any medical treatment<br>Agreement that the patient should keep a symptom diary for a limited period, explanation thereof

In the foreground	Understanding of symptoms, forming the therapeutic helping alliance
Elements	Information adapted to lead symptoms,<br>diary of symptoms, formulating the therapeutic target,<br>after second session introduction of relaxation training
Procedure	Developing a „map of somatic and psychic complaints“ including all relationship episodes related to the complaints (behaviour of doctors, relatives etc., experiences with former treatments) and related affects, experiences of not being taken seriously
	Actively asking what the patient thinks what the causes of the complaints are
	Actively asking about the current treatment expectations, questioning these if necessary in order to prevent disappointment
	Developing a sketch of the current life situation (including resources: social network, job-related stability etc.) and about biography (if known from former interviews, refer to that)
	Conveying information about psychophysiological contexts regarding the respective lead symptoms (instruction with the use of visual material and handouts for the patients)
	Formulating the targets of the intervention together („therapy target“) for follow-up and registering any change of targets in the therapy process. No standardised registration within the scope of the scientific investigation.
Tools	- Handing out information leaflets for patients about possible sociotherapeutic interventions (severe disability, retirement, rehabilitation, special integration service)<br>- The symptom/ context diary will be handed out at the end of the second and third session and will be talked about at the beginning of each session. A symptom diary will be used for a period of 2 weeks (intensity of the symptoms, impairment by the symptoms, mood on the relevant day, each evaluated on a scale between 0-10). A diary about the symptom contexts will be handed out in the next treatment sessions which additionally asks about psychosocial events.<br>- CD with PMR<br>- Explanation and handout of a diary for registering the contacts with doctors, other treatments (physiotherapy , massage) , and the use of medical services
Body-orientated work	Start of the use of relaxation elements early, i.e. depending on the individual situation in the first or second therapy session.<br>In any case introduction of PMR; where necessary alternatively introduction of an imaginative technique.<br>At the end of every session, 10 min. allowance for the guided relaxation or imagination technique
Homework	Relaxation exercises on a regular and daily basis, reading of the information, keeping the diaries

Therapy Phase 2

In the second phase, the focus will firstly lie on the patient‘s relationship to his/her own body and secondly on the emotions and relationship experiences. 5-6 sessions are provided for these topics.

**Relationship to one's own body**	
Topic	Symptom management
Elements	Clarification of the individual symptom context
Procedure	After accepting the symptom complaint (phase 1), enriching this complaint with one's own emotions and images;<br>Organising complaint, using linguistic symbols
	Counselling regarding body perception,<br>encouraging activity, collecting experiences with body-related activity (sports) and relaxation in order to experience bodily skills and limits in a new way (vitality, pleasure, lust, unpleasant bodily sensations) and in order to learn to rely more on physical capacities
	When bodily reactions occur, distinguishing between complaints and emotion, thought, evaluation and mostly automatized activities (according to symptom diary)
	Talking about the disappointments felt at one's own body because of lack of bodily functioning and the shame that is related to that, and pointing out the newly gained experiences with one's own body
Homework	Encouraging body-related activities, referring to previous hobbies (e.g. sports), regular relaxation exercises or regaining one's ability to enjoy life (cinema, theatre, etc.)

**Affects and relationship experiences**	
Topic affects	Supporting the perception and differentiation of emotions and bodily complaints or becoming aware of emotional participation, feeling ≠ thinking
Topic relationship	Importance of relationship experiences<br>Recording of correlations between the symptoms and the relationship experiences in current life, according to biography, in the therapeutic relationship
Elements	Integrating contexts of situation and relationship into the symptom complaint
Procedure	Evaluating and specifying occurring emotions in terms of „level of emotional awareness"; emotions regarding relationship experiences mostly in the health care system, relatives, therapist or in connection with the symptom experiences in order to clarify affects and relationships
	Supporting affect regulation in the relationship
	Treating the frustration aggression in different contexts:<br>frustration felt at one's own body / fighting against the symptom/ disappointed hope for help by medical staff / relationship break-ups<br>Frustration felt at behaviour of the others/ relatives/ the therapist
	Developing an alternative way of dealing with the affect of frustration
Problems	In case of a longer-lasting analysis of strains, conflicts or traumata regardless of the symptoms, it is important to explicitly clarify with the patient whether this happens by mutual agreement as this was not initially agreed upon.<br>If necessary, it should be pointed out that there is the possibility of a continuative therapy.
Structural Vulnerability	The use of the described attitudes and psychotherapeutic rules is also appropriate for patients with severe structural vulnerability. For them, the focus of affect clarification will yet lie more often on skills for self regulation and less on the aspect of relationship clarification.
Homework	Feed back on relaxing exercises. Keeping the diary about visits to doctor, change of medication, etc. , question about the information leaflet about social therapy

Therapy Phase 3

Taking stock	
Topic	**Ending and transfer**
Time	3 sessions
Elements	Developing strategies how to maintain achieved improvements, preventing a relapse
Procedure	Taking stock of the elements of phase 1-3, dealing with the termination/ limit- topic, actively stressing the things that were not achieved
	Tie in with the frustration topic of phase 2
	Anticipating (Planning/ Structuring) the time after the intervention in order to prevent a relapse<br>regarding: use of gained knowledge
	Planning further (psycho or social)therapeutic measures,<br>Planning further job-related and private activities
**Means**	Written summary of the things that were clarified (letter to the patient, e.g. regarding treatment of symptom / body, relatives, etc)<br>Written plan of possible changes in daily life<br>Specifying a concrete goal for the next 3 months on a special form
Tip	**Booster session after 3 months, making appointment now!**

Management of special aspects and problems

Parallel somatic treatments
Parallel to the psychosomatic interventions, the patients will often take further diagnostic and treatment measures, these are partly not even made transparent at the beginning. The intervention has a goal of setting a „new beginning“ in the disturbed relationship of the patient with the health care system and tries to encourage cooperation with the patient and with the co-treaters. Making somatic treatment measures transparent or possibly doing without them is mildly positively connotated by the therapist. Doing without parallel treatments is not demanded, but in cases of apparent (financial) self-damaging treatments a clear opinion is expressed if necessary. The family doctor will receive written information about the planned treatment within the study at the beginning thereof. Furthermore, the family doctor will be contacted if necessary during the intervention, but in any case at its ending.

Parallel pharmacologic treatments
The attitude on this is analogous: parallel medication, whether somatic, analgetic or e.g. antidepressive, will be accepted at the beginning, if necessary, it will be worked towards taking medicine according to plan and not according to need. Transparence and possible rejection are mildly connotated in a positive way, only in case of clearly not indicated drugs (e.g. opiates, benzodiazepines, fluspirilene) a clear opinion is expressed.

Repeated assurance that there is no or no sufficient organic reason to explain the complaints
Even though the intervention is not primarily aimed at patients with hypochondriasis in the stricter sense, it may happen in the course of the intervention that patients repeatedly urge the therapist to tell them that they do not have a severe illness „for reassurance“ and ask him to undertake further investigations. How to deal with this behaviour depends on the phase, on how to assess the stability of the helping alliance and how to assess the structural vulnerability of the patient.
Initially and in less stable situations, a respective assurance that they do not suffer from a severe illness might be helpful. Later on and in more stable situations, it might be agreed upon with the patient that there will be no assurance and he/she has to endure the triggered fear until it eases away. (It might be necessary to include the relatives into this agreement).

Management of „re-somatization“
During the course of a therapy, it happens almost regularly that distinctively somatoform patients increasingly complain about their symptoms and the feared organic reasons thereof, and all insights into the situative and affective contexts seem to be lost. In this situation, it is important to accept this re-somatized offer as currently necessary for the patient and not to confront the patient with the seemingly lost insights, and at worst in a disappointed and reproachful way. When the therapist maintains an accepting attitude, the patient will sooner be able to use these insights again.

Management of „superficial psychologization“
If the patients soon talk only about their intrapsychic and general relationship problems and spontaneously do not mention the symptoms and impairments anymore or do just seldom mention them, the therapist must actively ask about them, as there is the risk in this constellation that the psychologization stems from a latent loss of self esteem or subordination under the therapist and is not the consequence of a change of the symptom in the context of current life.

Management of impending drop-out
Owing to the described tendency for repeated frustration experiences in treatments, partially after a previous idealization of the therapist, and owing to the often low tolerance level of the patients to bear with these frustrations in the therapeutic relationship, it might happen very soon that the patient wants to stop the therapy. If the patient indicates that he/she wants to stop the therapy, the therapist should firstly discuss the advantages/disadvantages of an early ending with the patient, and not urge him to continue with the therapy. In doing so, the therapist should emphatically deal with the patient’s frustration and, together with the patient, develop hypotheses regarding affects and possibly real fears or fears based on transference and discuss them with the patient. Distinctively negative transferences should be actively talked about. In case the patient just misses the session, the therapist should write to the patient and suggest another appointment.

Management of missed appointments
At most 2 missed appointments can be made up for by additionally agreed appointments, regardless of the reason. In case of a missed appointment without excuse, this will be explicitly talked about regarding its meaning for the treatment in the next session.

References:

Henningsen P, Hartkamp N, Loew T, Sack M, Scheidt CE, Rudolf G: Somatoforme Störungen. Leitlinien und Quellentexte. Stuttgart: Schattauer 2002.

Wöller W, Kruse J (Hg.) Tiefenpsychologisch fundierte Psychotherapie. Basisbuch und Praxisleitfaden. Stuttgart: Schattauer 2001.

Rudolf G, Henningsen P: Die psychotherapeutische Behandlung somatoformer Störungen. Z Psychosom Med Psychother 49 (2003) .3-19.

Rief W, Henningsen P. Somatoforme Störungen. In Senf W, Broda M: Praxis der Psychotherapie 3. Aufl. Kap. X/36. Stuttgart: Thieme 2005, p 529-544.

Supplement 3b: Guideline for Control Group intervention

Enhanced Medical Care

General notes

In this trial, enhanced medical care is treatment as usual with additional definition of the appointment structure and the communicative strategies in dealing with the patient. The latter features should enhance homogeneity of treatment in the control group across trial sites and also enhance the likelihood of continuing patient participation.

In general, treatment in this arm of the study aims at alleviating pain-related lead symptoms and disabilities. It is performed by the physicians working in the university-based somatic outpatient treatment units that collaborate with the psychosomatic departments at the respective study sites.

The guideline encompasses three aspects: appointment structure, symptom-oriented measures and physician-patient communication. The physicians in charge of the respective somatic treatment unit will supervise the treatment. A workshop will be held with all physicians involved in the study to acquaint them in detail with the procedures.

Appointments

After being screened and interviewed either in a somatic inpatient or outpatient setting and after entering the study, patients in this study arm will have at least three appointments with the physician at the somatic outpatient treatment unit, e.g. a pain center. The respective appointments are six weeks apart at most. Additional appointments are possible, as needed.

- the emphasis of the first appointment is on negotiation of diagnosis and treatment plan
- the emphasis of the second appointment is on interim results and adjustment of treatment measures
- the emphasis of the third appointment is on planning and negotiating the future course of treatment

The amount of time spent during the appointments will vary according to clinical needs.
The patients will be encouraged, but not obliged to withhold treatment with other specialists at the same time.

Symptom-oriented measures

As type of lead symptoms and specific treatment setting vary between patients and sites, this guideline cannot be symptom-specific in the strict sense. However, physicians will follow the symptom-specific treatment guidelines published by their respective scientific society within the AWMF (working group of German scientific medical societies).
There are some common principles underlying the different guidelines for treating frequent symptoms like headache, back pain, muscular or lower abdominal pain.

These common principles guide treatment also in this arm of the study, they are as follows:

- competent exclusion of relevant organic causes underlying the symptoms
- treatment of underlying organic causes, when relevant
- rational alleviation of pain and other symptoms by pharmacologic means, including analgesics, but also other types of drugs like muscle relaxing agents, regulators of bowel movement frequency etc.
- additional use of physiotherapy where indicated
- additional use of psychotherapy where indicated
- encouragement of activation rather than rest
- change of therapeutic strategy in case of insufficient symptomatic success after a pre-defined period of time

The physicians taking part in this arm of the study are explicitly encouraged to use the same guideline-based principles they use in treating patients with these lead symptoms outside the study, including criteria for sending patients to psychotherapy.

Physician – patient communication

Definition of physician-patient communication aims at the following

- to avoid iatrogenic factors in the communication that potentially contribute to the persistence of symptoms and illness beliefs
- to ensure active cooperation of the patient in the treatment process
- to enhance homogeneity of treatment across study sites and physicians
- to enhance patient adherence to treatment

The following components should be included in communicating with the patient:

- information about the diagnoses, including the research diagnosis of multisomatoform disorder
- information about treatment alternatives, including self help strategies and complementary medicine
- enquiry about patient's treatment expectations
- negotiation of treatment decisions ("shared decision")

Principles of patient-centered care, including mutual respect and openness for differing views, should underlie these topics. The practice guideline "Defining the patient-physician relationship for the 21st century" (Johns Hopkins; American Healthways 2004) will be distributed and used as a common reference at all study sites.

References:

Arbeitsgemeinschaft der Wissenschaftlichen Medizinischen Fachgesellschaften (AWMF): Leitlinien-Datenbank. Online: http://leitlinien.net

Johns Hopkins; American Healthways. Defining the patient-physician relationship for the 21st century. Dis Manag 2004; 7: 161-179.

Supplement 4: Draft Patient information leaflets and consent forms

Note: The following information leaflets and consent forms for patients concern two different stages of the study, the participation in the Screening/ Assessment process and the participation in the study itself. The draft forms are given for one centre only, they are adapted to the local needs of the other centres. The information and consent form for the fMRI study is not enclosed.

Patient information leaflet, Screening Phase, English translation

University of Heidelberg
Dept. of Psychosomatics
In Co-operation with the
Pain Center at
University of Heidelberg

Patient information for completion of screening questionnaire and participation in interview on physical symptoms:

Dear Patient,

The Pain Center of Heidelberg University, in co-operation with the Department of Psychosomatics, carries out a study dealing with treatment of disorders not fully explainable on somatic grounds (somatoform disorder). This study is supported by the German research community and the Education Ministry.

In order to ascertain if you might be suitable for participation in such a study, we request that you complete a physical disorders questionnaire. We may then invite you to a brief discussion of your physical symptoms. Thereafter you may be asked if you would be agreeable to participate in a treatment study of your complaints. Discussion of these matters will require approximately 30 minutes of your time.

All data gathered shall be processed confidentially, remaining subject to full protection under relevant laws. Should you elect neither to complete the screening form, nor to participate in the resulting discussion, this will have no adverse consequences for you.

Thank you for your participation in this endeavour

Prof. H. Bardenheuer, M.D. N. Sauer, M.D.

Patient consent form, Screening Phase, English translation

University of Heidelberg
Dept. of Psychosomatics
In Co-operation with the
Pain Center at
University of Heidelberg

Name:

Internal Number:	
Date:	
Explained by:	

Declaration of Consent:

I hereby agree to completion of a questionnaire covering physical disorders, to possible participation in a related discussion of these physical disorders, and to explanation of a possible treatment study.

I am aware that I can rescind this consent without provision of grounds at any time and with no adverse consequences regarding my further medical-therapeutic treatment.

I have been instructed in these matters, and affirm that my name and all other confidential information are subject to the confidentiality rule of physicians, as well as the terms of German federal data-protection statutes (BDSG). Any further propagation of patient data may take place where appropriate only in anonymous form. Third parties are specifically excluded from access to original medical records.

I have received and read the explanation for patients. I have also been informed via consultation, in the course of which all of my questions were answered.

____
Place/ Date/ Signature

Patient information leaflet, Study Phase, English translation

University of Heidelberg
Dept. of Psychosomatics
In Co-operation with the
Pain Center at
University of Heidelberg

Patient information pertaining to the study:

„Psychosomatic Treatment of Somatoform Disorders – PISO“

Dear patient,

The Pain Center of Heidelberg University, in co-operation with the University Clinic for Psychosomatic Disorders, carries out a study dealing with treatment of disorders not fully explainable on normal, somatic grounds. This study is supported by the German Research Foundation and the Education Ministry.

Within the framework of this study the effectiveness of a newly developed treatment form is to be determined. This treatment form was developed specifically for patients whose disorders are of greater than two years' duration, for whose disorders to date no sufficient physical cause could be determined, and whose disorders could not be ameliorated by treatment so far.

<u>Description of study:</u>
All patients admitted to the study shall be assigned **at random** either to the new treatment measure group, or to a control (comparison) group. In other words, not all patients admitted to the study will be assigned to the new treatment measure.

The study treatment will last approximately four months for all patients. Those patients assigned to the new treatment measure shall have, in addition to the regular treatment offered, approximately 12 appointments of about 50 minutes' duration each in the Pain Center, and one appointment of about 60 minutes' duration after one year.

Regular participation in sessions is essential for the success of the study. The **control (comparison) group** will receive the regular treatment offered by the Pain Center, in enhanced form: that is, with three monthly regular and well-established

evaluation appointments pinned to the overall treatment regimen, but without the special treatment measures. In addition, there will be two further appointments after three months and after one year.

The **new treatment** method consists of the following parts:

Part one of the sessions: improved understanding of the disorder(s):
- Thorough anamnesis of disorder(s)
- Provision of information regarding origin, increasing versus diminishing gravity of disorder(s),
- Acquisition of new relaxation methods

Part two of the sessions: dealing better with disorder(s)
- Development of capabilities which do not place disorder(s) in the centerpoint of the daily routine
- Improvement of perception of bodily signals
- Improvement of perception of difficulties in dealing with disorder(s), including relations with care-providers and family members
- Concrete, feasible, individual suggestions aimed at gradual increase of activity and re-introduction into light physical and/or athletic activities

Part three of the sessions: application of new insights to daily use
- Strategies for maintenance of improvements
- Relapse prophylaxis
- Planning of concrete, feasible and individual measures

The programme thus consists of a treatment regimen designed to gather new insights into physical disorders, which in turn help reduce the impact of such disorder(s), and facilitate a renewed and expanded range of activity and movement – by resumed interest in former hobbies, for example.

Initial studies show that such treatment regimens, when pursued vigorously, lead to the manner of measurable and enduring improvements articulated above.

Measurement of treatment success is carried out using questionnaires; but also by measuring the natural fluctuation in heart rhythm, with a view to establishing stress-management capacity.

<u>Summary of heart frequency measurement:</u>

The more pronounced the fluctuation in a person's heart rhythm, the greater is that individual's adaptability, as well as his or her capacity to cope with stressful situations. Heart frequency is recorded by means of a simple transmitter strapped around the chest. The transmitter sends EKG signals in wireless mode to a PC. There is absolutely no danger of electrical shock. The product bears a CE safety seal of approval in accordance with Euro-Norm 93/94.

What can you expect as a potential study participant?

- All patients will receive questionnaires from us, each of which can be filled out in about 20 minutes. You will receive these forms at the beginning of the study, after the first six weeks, after completion of treatment, and once again after 12 months.
- All patients will be tested for heart rhythm fluctuation, in order to establish ability to cope with stress. These measurements will be carried out in sessions of roughly 20 minutes in duration: at the beginning of the study, after 12 weeks, and after 12 months.
- For those patients assigned to the treatment programme, this means that in addition to the questionnaires, there will be a time expenditure of 12 fifty-minute sessions for psychosomatic treatment.

This offer is intended to provide future support in improved management of your disorder(s). For treatment group participants this might lead to temporary depression as a side effect. This would be taken into account in the course of the psychosomatic treatment itself.
Through your participation in this study, you help us improve therapeutic options for patients with somatoform disorders. This will be of benefit both to you today and to other patients with similar disorders in future.

Should you choose not to take part in the study, there will be no adverse consequences for you. You will be given the regular treatment provided by the Pain Center, albeit under exclusion from participation in the programme described above.

Your personal medical data are naturally protected both by the rule of confidentiality for physicians and German data-protection laws. Scientific analysis will take place in a climate of anonymity, e.g., without mention of patients' names or the possibility of deducing those names by third parties. Participation in this examination is of course voluntary. You may decline at any time, with no adverse effects on your therapeutic care. Should you elect to discontinue, we will again request your permission to use data already acquired through your participation to that point

Should you have any further questions, please do not hesitate to contact us at one of the addresses listed below.

We thank you for your assistance.

Prof. Bardenheuer, M.D.
Director, Pain Center

Private Lecturer Henningsen, M.D.
Deputy head, Dept. of Psychosomatics

Dr. Sauer, M.D.
University Dept for Psychosomatic Disorders

Patient consent form, Study Phase, English translation

University of Heidelberg
Dept. of Psychosomatics
In Co-operation with the
Pain Center at
University of Heidelberg

Name:

Internal Number:	
Date:	
Explained by:	

Declaration of Consent:

Regarding participation in the Study:

„Psychosomatic Treatment of Somatoform Disorders - PISO“

possibly resulting in random assignment to one of two separate therapy measures in the course of this study

I agree to participate in the study „Psychosomatic Treatment in Somatoform Disorders – PISO“. I have been informed that I will be assigned at random, either to the new or customary treatment regimen. I am aware that I can rescind this consent without provision of grounds at any time and with no adverse consequences regarding my further medical-therapeutic treatment.

I have been instructed in these matters, and affirm that my name and all other confidential information are subject to the confidentiality rule of physicians, as well as the terms of German federal data-protection statutes (BDSG). Any further propagation of patient data may take place where appropriate only in anonymous form. Third parties are specifically excluded from access to original medical records.

In the event of my discontinued participation in this study, I consent to the evaluation of data resulting from participation up to that point.

Yes ❍ No ❍

I have received and read the explanation for patients. I have also been informed via consultation, in the course of which all of my questions were answered.

____
Place/ Date/ Signature

Supplement 5: Methods for Psychophysiological Measurements

Autonomic measurement
All patients both from the treatment and the control group will be assigned to psychophysiological assessment during a stress test before and after the intervention period. Pre-test as well as post-test anxiety and stress levels will be assessed using a rating of subjective units of discomfort (SUD) on a scale ranging from 0 to 10 (Wolpe 1969). After attachment of the ECG chest-belt (Polar S810) the Stroop-test will be explained to the patients followed by a 2-min training period. Pre-test stress-levels and anxiety will be assessed. Then 5 min of rest without any task will follow where the last 2 minutes will serve as Baseline 1. Stroop test will be applied with duration of 5 minutes followed by a recovery period of 3 minutes. Post-test stress-levels and anxiety will be assessed by SUD ratings immediately after the recovery period Another 5 minutes of rest follow where the last 2 minutes will serve as Baseline 2.

A time series of inter-beat intervals with a time-resolution of 1000 Hz will be generated from the wireless transmitted ECG signal. HR will be calculated as a mean value for each measuring condition of 2 min duration. HRV will be calculated both in time domain (RMSSD) as well as in frequency domain (HF-HRV) according to published standards (Task Force of the European Society of Cardiology and the North American Society of Pacing and Electrophysiology 1996). HRV-values will be statistically controlled for age, body mass index and for breathing frequency as derived from peak HF-frequency. Due to the expected skewness of data, RMSSD will be logarithmically transformed for statistical comparison. HR and HRV reactivity to Stroop-test will be calculated by subtracting measures during Stroop-test levels with the mean of baseline 1 and baseline 2.

A repeated measures analysis of variance (ANOVA) model with the between subjects factor group (treatment vs. control group), the within subjects factor time and the covariates sex and age will be used for examination of HR and HRV during baseline, stress test and for reactivity scores.

Supplement 6: References

Bagby RM, Parker JD, Taylor GJ The twenty-item Toronto Alexithymia Scale--I. Item selection and cross-validation of the factor structure. J Psychosom Res. 1994 Jan; 38(1):23-32.
Barsky AJ, Borus JF. Functional somatic syndromes. Ann Intern Med. 1999;130:910-21.
Bass C, Hyde G, Bond A, Sharpe M. A survey of frequent attenders at a gastroenterology clinic. J Psychosom Res 2001; 50: 107-109.
Bassler M, Potratz B, Krauthauser H. Der „Helping Alliance Questionnaire" (HAQ) von Luborsky. Psychotherapeut (1995) 40: 23-32
Brennan K, Clark C, Shaver, P. Self-report measurement of adult attachment. An integrative overview. In Simpson J & Rholes W (Eds.). Attachment theory and close relationships (pp. 46-76). New York (1998): Guilford.
Broadbent E, Petrie KJ, Main J, and Weinman J. The Brief Illness Perception Questionnaire (BIPQ). Health Psychology (in submission).
Bullinger M, Kirchberger I. SF-36 - Fragebogen zum Gesundheitszustand. Göttingen: Hogrefe, 1998.
Chambers AS, Allen JJ. Vagal tone as an indicator of treatment response in major depression. Psychophysiology 2002; 39: 861-864.
Christensen KS, Toft T, Frostholm L, Ornbol E, Fink P et al.. The FIP study: a randomised, controlled trial of screening and recognition of psychiatric disorders. Brit J Gen Pract 2003; 53:758-63
Cohen H, Neumann L, Kotler M, Buskila D. Autonomic nervous system derangement in fibromyalgia syndrome and related disorders. Isr.Med Assoc.J 2001; 3: 755-760.
Creed F, Lakshmi F, Guthrie E, et al. The cost effectiveness of psychotherapy and Paroxetine for severe irritable bowel syndrome. Gastroenterology 2003: 124:303-317
Dickinson WP, Dickinson LM, deGruy FV, et al. The somatization in primary care study: a tale of three diagnoses. Gen Hosp Psychiatry 2003; 25: 1-7.
Fuchs T. Neurobiology and psychotherapy: an emerging dialogue. Curr Op Psychiatry 2004;17:479-85
Gaab J: Psychometrische Evaluation des Illness Perception Questionnaires (IPC-R) im deutsch-sprachigen Raum. Unpublizierter Forschungsbericht Universität Zürich 2003.
Gallati D. Metaanalysis on measurement of treatment success in trials on depression treatment. Unpublished thesis, Institute of Psychology, University Bern, Switzerland.
Grabe HJ, Meyer C, Hapke U et al. Somatoform pain disorder in the general population. Psychother Psychosom 2003; 72: 88-94.
Gündel H, Valet M, Sorg C, et al.. Hypoactivity of ventromedial prefrontal cortex in patients with somatoform pain disorder - a controlled fMRI study. Psychosomatic Medicine, in press (2005).
Gureje O, Simon GE. The natural history of somatization in primary care. Psychol Med 1999; 29: 669-676.
Guthrie E, Moorey J, Margison F et al. Cost-effectiveness of brief psychodynamic-interpersonal therapy in high utilizers of psychiatric services. Arch Gen Psychiatry 1999; 56: 519-526.
Hamilton J, Guthrie E, Creed F et al. A randomized controlled trial of psychotherapy in patients with chronic functional dyspepsia. Gastroenterology 2000; 119: 661-669.
Henningsen P, Hartkamp N, Loew T, Sack M, Scheidt CE. Somatoforme Störungen - Leitlinien und Quellentexte [Somatoform disorders: guidelines and source texts]. Stuttgart: Schattauer, 2002.
Henningsen P, Zimmermann T, Sattel H. Medically unexplained physical symptoms, anxiety, and depression: a meta-analytic review. Psychosom Med 2003; 65: 528-533.
Henningsen P, Jakobsen T, Schiltenwolf M, Weiss MG. Somatisation revisited: diagnosis and perceived causes in common mental disorders. Journal of Nervous and Mental Disease 2005; 193:1-8
Kroenke K, Spitzer RL, deGruy FV et al. Multisomatoform disorder. An alternative to undifferentiated somatoform disorder for the somatizing patient in primary care. Arch.Gen.Psychiatry 1997; 54: 352-358.
Leichsenring F, Rabung S, Leibing E. The efficacy of short-term psychodynamic psychotherapy in specific psychiatric disorders: a meta-analysis. Arch Gen Psychiatry 2004; 61:1208-16.
Löwe B, Unutzer J, Callahan CM, Perkins AJ, Kroenke K. Monitoring depression treatment outcomes with the patient health questionnaire-9. Med Care 2004; 42:1194-201
Luborsky L. Principles of psychoanalytic psychotherapy. A manual for supportive-expressive psychotherapy. Basic Books (1984), New York
Margison F. Psychodynamic-interpersonal therapy. Oxford: Oxford University Press, 2002.
Nanke A, Rief W. Zur Inanspruchnahme medizinischer Leistungen bei Patienten mit somatoformen Störungen [Health care utilisation in patients with somatoform disorders]. Psychotherapeut 2003; 48: 329-335.
Neumann E, Rohmann E, Bierhoff HW. Development and validation of scales for measuring avoidance and anxiety in relationships – The Bochum Adult Attachment Questionnaire. Submitted

Nimnuan C, Hotopf M, Wessely S. Medically unexplained symptoms: an epidemiological study in seven specialities. J Psychosom Res 2001; 51: 361-367.
Reid S, Crayford T, Patel A, Wessely S, Hotopf M. Frequent attenders in secondary care: a 3-year follow-up study of patients with medically unexplained symptoms. Psychol Med 2003; 33: 519-524.
Reid S, Wessely S, Crayford T, Hotopf M. Medically unexplained symptoms in frequent attenders of secondary health care: retrospective cohort study. BMJ 2001; 322: 767.
Rief W, Henningsen P. Somatoforme Störungen [Somatoform Disorders]. In Senf W, Broda M (ed.): Praxis der Psychotherapie 3rd ed. Ch. X/36. Stuttgart: Thieme 2005, p 529-544.
Rief W, Hiller W. A new approach to the assessment of the treatment effects of somatoform disorders. Psychosomatics 2003; 44:492-8.
Rudolf G, Henningsen P. Die psychotherapeutische Behandlung somatoformer Störungen [Psychotherapeutic treatment of somatoform disorders]. Z Psychosom Med Psychotherap 2003; 49: 3-19
Startup M, Shapiro DA. Therapist treatment fidelity in prescriptive vs. exploratory psychotherapy. Br J Clin Pychol. 1993; 32: 443-56.
Stewart W, Schwartz B, Ricci J. Health-related workplace productivity measurement: adding to the list. J Occup.Environ.Med 2003; 45: 912-914.
Thayer JF, Lane RD. A model of neurovisceral integration in emotion regulation and dysregulation. J Affect.Disord 2000; 61: 201-216.
Waller E, Scheidt CE. Somatoform disorders as disorders of affect regulation: a study comparing the TAS-20 with non-self-report measures of alexithymia. J Psychosom Res. 2004;57 :239-47.
Ward E, King M, Lloyd M, et al. Randomised controlled trial of non-directive counselling, cognitive-behaviour therapy, and usual general practitioner care for patients with depression. I: clinical effectiveness. BMJ. 2000;321:1383-8
Wessely S, Nimnuan C, Sharpe M. Functional somatic syndromes: one or many? Lancet 1999; 354: 936-939.
Wiebe S, Matijevic S, Eliasziw M, Derry PA.Clinically important change in quality of life in epilepsy. J Neurol Neurosurg Psychiatry 2002; 73: 116-120
